# *Giardia duodenalis* Induces Proinflammatory Cytokine Production in Mouse Macrophages *via* TLR9-Mediated p38 and ERK Signaling Pathways

**DOI:** 10.3389/fcell.2021.694675

**Published:** 2021-07-15

**Authors:** Xudong Pu, Xin Li, Lili Cao, Kaiming Yue, Panpan Zhao, Xiaocen Wang, Jianhua Li, Xichen Zhang, Nan Zhang, Zhiteng Zhao, Min Liang, Pengtao Gong

**Affiliations:** ^1^Key Laboratory of Zoonosis Research, Ministry of Education, College of Veterinary Medicine, Jilin University, Changchun, China; ^2^Department of Parasite, Jilin Academy of Animal Husbandry and Veterinary Medicine, Changchun, China

**Keywords:** *Giardia duodenalis*, TLR9, p38, ERK, cytokines

## Abstract

*Giardia duodenalis*, also known as *Giardia lamblia* or *Giardia intestinalis*, is an important opportunistic, pathogenic, zoonotic, protozoan parasite that infects the small intestines of humans and animals, causing giardiasis. Several studies have demonstrated that innate immunity-associated Toll-like receptors (TLRs) are critical for the elimination of *G. duodenalis*; however, whether TLR9 has a role in innate immune responses against *Giardia* infection remains unknown. In the present study, various methods, including reverse transcriptase–quantitative polymerase chain reaction, Western blot, enzyme-linked immunosorbent assay, immunofluorescence, inhibitor assays, and small-interfering RNA interference, were utilized to probe the role of TLR9 in mouse macrophage-mediated defenses against *G. lamblia* virus (GLV)–free or GLV-containing *Giardia* trophozoites. The results revealed that in *G. duodenalis–*stimulated mouse macrophages, the secretion of proinflammatory cytokines, including interleukin 6 (IL-6), tumor necrosis factor α (TNF-α), and IL-12 p40, was enhanced, concomitant with the significant activation of TLR9, whereas silencing TLR9 attenuated the host inflammatory response. Notably, the presence of GLV exacerbated the secretion of host proinflammatory cytokines. Moreover, *G. duodenalis* stimulation activated multiple signaling pathways, including the nuclear factor κB p65 (NF-κB p65), p38, ERK, and AKT pathways, the latter three in a TLR9-dependent manner. Additionally, inhibiting the p38 or ERK pathway downregulated the *G. duodenalis*–induced inflammatory response, whereas AKT inhibition aggravated this process. Taken together, these results indicated that *G. duodenalis* may induce the secretion of proinflammatory cytokines by activating the p38 and ERK signaling pathways in a TLR9-dependent manner in mouse macrophages. Our *in vitro* findings on the mechanism underlying the TLR9-mediated host inflammatory response may help establish the foundation for an in-depth investigation of the role of TLR9 in the pathogenicity of *G. duodenalis*.

## Introduction

*Giardia duodenalis*, also known as *Giardia lamblia* and *Giardia intestinalis*, is an opportunist protozoan parasite that predominantly parasitizes the duodenum of humans, as well as of numerous domestic and wild animals, causing giardiasis. The disease is distinguished by a broad spectrum of clinical manifestations ranging from asymptomatic infection to vomiting, abdominal pain, weight loss, severe diarrhea, and malabsorption syndrome. Giardiasis is one of the most common pathogenic parasite infections in humans, with approximately 280 million cases of symptomatic giardiasis being reported annually worldwide (Einarsson et al., 2016). Giardiasis has been included in the World Health Organization’s neglected disease initiative since 2004 (Savioli et al., 2006), owing to its severe impact on children, which includes severe malnutrition, physical retardation, and poor cognitive function (Berkman et al., 2002). In addition, giardiasis has been reported to the Centers for Disease Control and Prevention (CDC) of the United States since 1992 and became a nationally notifiable disease in 2002 (Coffey et al., 2021). According to the CDC, because of poverty, poor drinking water quality, and limited treatment options, nearly 33% of the population in developing countries is afflicted with giardiasis, as is 2% of the adult population in the developed world (Kunz et al., 2017). *Giardia* infection has clearly become a non-negligible problem, attracting widespread research attention from an increasing number of scientists.

*Giardia lamblia* virus (GLV), first identified in 1986 (Wang and Wang, 1986), is a double-stranded RNA virus in the family *Totiviridae* that specifically infects trophozoites of *G. duodenalis* (Lagunas-Rangel et al., 2021). GLV is the only one of the known protozoal dsRNA viruses that can transmit efficiently by extracellular means, although the pathway involved is not well outlined (Wang and Wang, 1986; Miller et al., 1988). Similarly, several other protozoan parasites, including *Leishmania*, *Trichomonas*, and *Cryptosporidium*, also harbor small dsRNA viruses, and several studies have reported that these viruses can enhance the pathogenicity of their respective protozoa and thus exacerbate the disease (Wang et al., 1987; Jenkins et al., 2008; Ives et al., 2011; Fichorova et al., 2012, 2013). Although an early review found no significant correlation between GLV and *Giardia* virulence (Wang and Wang, 1991), it is currently unclear whether GLV affects *Giardia* infectivity.

Innate immunity and adaptive immunity, two main components of the immune system, are crucial for the eradication of *Giardia* infection (Fink and Singer, 2017). Innate immunity acts as the first line of defense against infections by pathogenic microorganisms. The mammalian innate immune system mainly serves to recognize pathogen-associated molecular patterns (PAMPs) found in viruses, bacteria, fungi, and parasites but absent in mammalian cells, through corresponding pattern recognition receptors (PRRs) (Vadillo and Pelayo, 2012), which, in turn, initiate the relevant immune responses. Toll-like receptors (TLRs) are an ancient family of innate immune receptors and play a significant role in resisting parasite infections (Gay and Gangloff, 2007). When TLRs recognize parasite-derived PAMPs, they can activate downstream regulatory factors, such as nuclear factor κB (NF-κB) and interferon regulatory factor (IRF), which regulate immune-related signal transduction and induce the transcription and expression of proinflammatory factors, interferons, and chemokines, among other factors. They can also initiate innate and adaptive immune responses targeting parasite infection through the regulation of antigen-presenting cells (Takeda and Akira, 2004).

TLR9 is known to specifically recognize unmethylated cytosine–phosphate–guanine (CpG) motifs present in bacterial and viral DNA, thereby eliciting innate immune responses (Kumagai et al., 2008; Yasuda et al., 2009; Wu and Kuo, 2015), whereas TLR3, which also recognizes nucleic acids, chiefly recognizes the dsRNA genome from viruses (Vercammen et al., 2008; Tatematsu et al., 2014). Additionally, CpG motifs in parasites such as *Leishmania* have been found to act as ligands for TLR9 (Gupta et al., 2015), and dsRNA genome of *Leishmaniavirus* can be recognized by the host’s TLR3 (Ives et al., 2011), whereas *Leishmania* parasites that do not carry *Leishmaniavirus* cannot express dsRNA for TLR3 recognition (Franco et al., 2017). TLR9 activation has been shown to promote a host-protective response in *Leishmania*-infected mouse macrophages (Srivastava et al., 2013). Ligand recognition by TLR9 results in the activation of the mitogen-activated protein kinase (MAPK), AKT, and NF-κB signaling pathways (Xu et al., 2003; Das et al., 2015), which promotes the secretion of proinflammatory cytokines (Gazzinelli and Denkers, 2006; Kawai and Akira, 2009). Macrophages are a key component of the innate immune system, and many PRRs, such as TLRs, localize to either the cell membrane (TLR1, TLR2, TLR4, TLR5, and TLR6) or endosomes (TLR3, TLR4, TLR7, TLR8, and TLR9) of these cells (Park et al., 2011; Cui et al., 2014; Liu et al., 2017). Activated macrophages can produce a wide array of cytokines, including interleukin 6 (IL-6), tumor necrosis factor α (TNF-α), and IL-12 p40, which initiate the inflammatory response (Sodhi and Pandey, 2011). Furthermore, it has been demonstrated that mouse peritoneal macrophages (PMφs) infected with *G. duodenalis* secrete large amounts of these proinflammatory cytokines (Li et al., 2017). However, whether TLR9 plays a role in *G. duodenalis*–induced host macrophage-mediated inflammation and cytokine secretion, whether it plays a protective part in promoting host cytokine secretion or exacerbates disease progression or whether TLR9 has differential roles in host macrophages infected with GLV-free *Giardia* trophozoites and those infected with GLV-containing *Giardia* trophozoites remains unknown.

In the present study, we undertook an *in vitro* analysis of the role of TLR9 in the inflammatory response of mouse macrophages mediated by GLV-free and GLV-containing *Giardia* trophozoites and sought to identify the signaling pathways involved in this process. We found that, compared with GLV-free *Giardia* trophozoites, those containing GLV induced a stronger inflammatory response in mouse macrophages. We further found that TLR9 was significantly activated in mouse macrophages with *G. duodenalis* stimulation, an effect that involved proinflammatory cytokine production mediated by the TLR9–p38/ERK signaling pathways. These findings may establish the foundation for further research on the role of TLR9 in the pathogenicity of *G. duodenalis*.

## Materials and Methods

### Cultivation of *G. duodenalis* Trophozoites and Separation of Mouse PMφs

*Giardia lamblia* virus-free *Giardia* trophozoites were derived from the *G. duodenalis* WB strain (ATCC30957; American Type Culture Collection, Manassas, VA, United States), and GLV-containing *Giardia* trophozoites were derived from *G. duodenalis* Assemblage A1 preserved in the parasite laboratory of College of Veterinary Medicine, Jilin University (Gong et al., 2020). GLV-free and GLV-containing *Giardia* trophozoites were both cultivated for 48 h in modified TYI-S-33 medium. To enrich macrophages, wild-type (WT) female C57BL/6 mice (6–8 weeks old) were intraperitoneally injected with 3 mL of sterile 2.98% Difo fluid thioglycolate medium (Becton, Dickinson and Company, Sparks, MD, United States) (Murphy et al., 2017). Four days later, the mice were euthanized by an overdose of diethyl ether and then sterilized by immersion in 75% ethanol for 10 min (Li et al., 2017). The mouse peritoneal cavity was rinsed twice with 16 mL of sterile phosphate-buffered saline (PBS; pH 7.4), and separated PMφs were collected by centrifugation at 1,000 × *g* for 10 min and then washed twice with 16 mL of sterile PBS. A total of 3 × 10^6^ cells were incubated per well of 6-well culture plates (JET BIOFIL, Guangzhou, China) in 1 mL of RPMI 1640 medium supplemented with 10% fetal bovine serum (FBS), 2 mM L-glutamine, 100 U/mL penicillin, and 100 mg/mL streptomycin at 37°C with 5% CO_2_. Unattached cells were removed, and fresh medium was added before stimulation. All animal experiments were performed strictly according to the Regulations for the Administration of Affairs Concerning Experimental Animals approved through the State Council of the People’s Republic of China (1988.11.1) and with the approval of the Animal Welfare and Research Ethics Committee at Jilin University (IACUC permit no. 20160612). The C57BL/6 mice were purchased from Changsheng Experimental Animal Centre (Anshan, China) and were housed in filter-top cages in an air-conditioned animal facility in the National Experimental Teaching Demonstration Center of Jilin University (Changchun, China). Water and normal mouse food were provided *ad libitum*.

### Extraction of Genomic DNA (gDNA) From *G. duodenalis* Trophozoites

*Giardia* gDNA was extracted from GLV-free *Giardia* trophozoites using the TIANamp Genomic DNA Kit (Tiangen, Beijing, China). GLV-free *Giardia* trophozoites were collected by centrifugation at 1,000 × *g* for 10 min and washed three times with sterile PBS. GLV-free *Giardia* trophozoites (1 × 10^6^ per sample) were digested with proteinase K for 1 h, following which gDNA was extracted according to the manufacturer’s instructions. The concentration of gDNA was measured by UV absorbance at 260 nm using a Nanodrop (Thermo Fisher Scientific, Waltham, MA, United States).

### Analysis of TLR9 Gene Expression by Reverse Transcriptase-Quantitative Polymerase Chain Reaction

A total of 3 × 10^6^ WT PMφs were stimulated with 1 × 10^6^ *G. duodenalis* trophozoites for different times (2, 4, 6, 8, and 12 h). Unstimulated PMφs served as a blank control, whereas PMφs stimulated with 5 μM/mL CpG ODN 1668 (a murine TLR9 ligand; InvivoGen, San Diego, CA, United States) served as a positive control. Subsequently, 3 × 10^6^ WT macrophages were stimulated with *Giardia* gDNA (3 μg of gDNA extracted from 1 × 10^6^ GLV-free *Giardia* trophozoites), 1 × 10^6^ GLV-free *Giardia* trophozoites, 1 × 10^6^ GLV-containing *Giardia* trophozoites, or 5 μM/mL CpG ODN 1668 in 1 mL of RPMI 1640 medium containing 1% FBS, 2 mM L-glutamine, 100 U/mL penicillin, and 100 mg/mL streptomycin at 37°C with 5% CO_2_. After treatment, the supernatants of the cell cultures were discarded, and total RNA was extracted from mouse macrophages using TRIzol reagent (Invitrogen, Carlsbad, CA, United States). Total RNA was reverse-transcribed into cDNA with oligo(dT) primers using the PrimeScript 1st Strand cDNA Synthesis Kit (Takara, Dalian, China). The TLR9 mRNA level was normalized to that of β-actin. For polymerase chain reaction (PCR) amplification, the conditions were as follows: 95°C for 3 min, followed by 44 cycles of 95°C for 30 s, 56°C for 30 s, and 72°C for 30 s, with a final extension at 72°C for 10 min. Melting curves were analyzed following the PCR run to validate the homogeneity of PCR products. All the primers were synthesized by Sangon (Shanghai, China) and contained the following sequences: TLR9, 5′-CTGCCCAAACTCCACACTCT-3′ forward primer and 5′-ACAAGTCCACAAAGCGAAGG-3′ reverse primer (Zhao et al., 2021); and β-actin, 5′-TGCTGTCCCTGTATGCCTCT-3′ forward primer and 5′-GGTCTTTACGGATGTCAACG-3′ reverse primer (Li et al., 2017).

### SiRNA-Mediated Knockdown Assay

The TLR9-specific small-interfering RNA (siTLR9) (target sequence: 1#, 5′-GGAACTGCTACTACAAGAA-3′; target sequence 2#, 5′-CCTTCGTGGTGTTCGATAA-3′; and target sequence 3#, 5′-CCTATAACCTCATTGTCAA-3′) and scramble negative control of small-interfering RNA (siRNA) (siNC) were synthesized by RiboBio (Guangzhou, China). A total of 3 × 10^6^ WT PMφs were incubated per well of a 6-well culture plate, following which adherent cells were transfected with the indicated siRNAs using Lipofectamine 2000 (Invitrogen). Lipofectamine 2000 and siRNA were separately diluted in serum-free and antibiotic-free Opti-MEM medium, separately incubated at room temperature for 5 min, mixed and incubated for 15 min at room temperature, and finally added to the cells. After 6 h, the transfection medium was discarded; the cells were washed twice with RPMI 1640 medium and maintained in 1 mL of RPMI 1640 medium plus 2% FBS for another 24 h at 37°C with 5% CO_2_. The cells were incubated with 1 × 10^6^ GLV-free *Giardia* trophozoites or GLV-containing *Giardia* trophozoites for 18 h after transfection. The medium and bottom-layer cells were harvested separately for enzyme-linked immunosorbent assay (ELISA) and Western blot, respectively.

### Analysis of Cytokine Levels by ELISA

A total of 3 × 10^6^ WT or siTLR9-treated PMφs were stimulated with 1 × 10^6^ GLV-free *Giardia* trophozoites, 1 × 10^6^ GLV-containing *Giardia* trophozoites, 3 μg of *Giardia* gDNA, 5 μM/mL CpG ODN 1668, or 100 ng/mL lipopolysaccharide (LPS) (*Escherichia coli* 0111: B4; Sigma-Aldrich, St. Louis, MO, United States) for 18 h in 1 mL of RPMI 1640 medium containing 1% FBS at 37°C with 5% CO_2_ (Murphy et al., 2017; Li et al., 2019). The collected supernatants were temporarily stored at −80°C for ELISA. Cytokine concentrations in the supernatants were determined by uncoated ELISA kits specific for mouse IL-6 (88–7064), TNF-α (88–7324), and IL-12/IL-23 (total p40) (88–7120, all from eBioscience, San Diego, CA, United States) following the manufacturer’s instructions.

### Western Blotting

A total of 3 × 10^6^ WT PMφs were stimulated with 1 × 10^6^ *G. duodenalis* trophozoites for different times (0.5, 1, 2, 3, 4, 5, and 6 h) with unstimulated PMφs serving as a blank control. Following stimulation, the medium was collected, and the bottom-layer cells were scraped with a cell scraper, followed by centrifugation at 12,000 × *g* for 30 min at 4°C. The harvested cells were lysed with RIPA buffer (BOSTER, Wuhan, China) containing protease/phosphatase inhibitors (Sangon, dilution 1/100). Protein was quantified by bicinchoninic acid assay. Equal amounts (30 μg) of protein from the different samples were separated by 10% sodium dodecyl sulfate–polyacrylamide gel electrophoresis and then transferred to polyvinyl difluoride membranes (Millipore, Billerica, MA, United States). After blocking with 5% bovine serum albumin (BSA) in Tris-buffered saline 0.1 Tween 20 (TBST) for 2 h at room temperature, the membranes were incubated overnight at 4°C with primary antibodies targeting TLR9, p38, ERK, AKT, NF-κB p65, phospho-p38, phospho-ERK, phospho-AKT, phospho-p65, phospho-IκBα, β-actin (all rabbit), and IκBα (mouse) (all from Cell Signaling Technology, Danvers, MA, United States) diluted 1/1,000 in 5% BSA. The next day, the membranes were washed three times with TBST, incubated with secondary horseradish peroxidase–conjugated goat anti-rabbit immunoglobulin G (IgG) or anti–mouse IgG antibodies (Proteintech, Wuhan, China), diluted 1/5,000 in 5% non-fat milk for 45 min at room temperature, and then washed again three times with TBST. Protein bands were detected by enhanced chemiluminescence (Vigorous, Beijing, China). The protein expression level was densitometrically quantified using ImageJ.

### Analysis of NF-κB p65 by Immunofluorescence

A total of 7.5 × 10^5^ WT and siTLR9-treated PMφs were cultivated in 24-well culture plates on sterile glass coverslips and stimulated with 7.5 μg of *Giardia* gDNA, 2.5 × 10^5^ GLV-free *Giardia* trophozoites, 2.5 × 10^5^ GLV-containing *Giardia* trophozoites, or 5 μM/mL CpG ODN 1668 at 37°C with 5% CO_2_. After incubation for 60 min, the cells were washed three times with sterile PBS, fixed in 4% formaldehyde solution for 20 min at room temperature, washed three times with sterile PBS, permeabilized with 0.25% Triton X-100 for 20 min, washed three times, blocked in 3% BSA in PBST for 1 h at room temperature, and incubated overnight at 4°C with rabbit anti–NF-κB p65 antibody (Cell Signaling Technology) diluted 1/1,000 in 1% BSA. After washing three times, the cells were incubated with fluorescein isothiocyanate–conjugated goat anti–rabbit IgG (H + L) antibody (Proteintech) diluted 1/100 in 1% BSA for 1 h at room temperature, washed again three times, and then counterstained with DAPI at room temperature for 20 min. NF-κB p65 localization was visualized under a confocal microscope (LSM-710, Carl Zeiss, Oberkochen, Germany) equipped with a 63×, 1.4-NA, oil-immersion objective.

### Inhibition Assay

Wild-type PMφs (3 × 10^6^) were pretreated with the p38 inhibitor SB203580 (30 μM), ERK inhibitor PD98059 (40 μM), or AKT inhibitor MK-2206 2HCl (5 μM) (all from Selleck, Shanghai, China) for 30 min at 37°C with 5% CO_2_, followed by coincubation with 1 × 10^6^ GLV-free *Giardia* trophozoites or 1 × 10^6^ GLV-containing *Giardia* trophozoites in 1 mL of RPMI 1640 medium supplemented with 1% FBS. The medium and bottom-layer cells were separately harvested for ELISA and Western blot analysis.

### Statistical Analysis

Data were expressed as means ± SD from three separate experiments. GraphPad Prism 8 (GraphPad Software Inc., La Jolla, CA, United States) was utilized for ELISA data analysis. SPSS version 19.0 (SPSS Inc., Chicago, IL, United States) was employed for statistical analysis. Unpaired *t* tests were used to compare data between two groups, whereas one-way analysis of variance followed by Tukey test was used to compare data between multiple groups. *p* < 0.05 was considered statistically significant.

## Results

### *G. duodenalis* Trophozoites Induced TLR9 Activation and Proinflammatory Cytokine Production in PMφs

**FIGURE 1:**
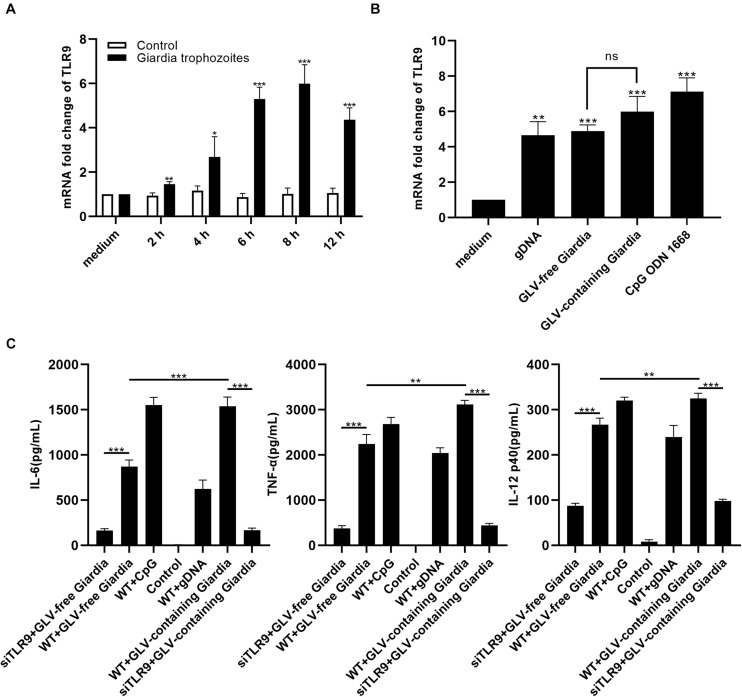
*Giardia duodenalis* trophozoites induced cytokine production in a TLR9-dependent manner. **(A)** RT-qPCR analysis of the relative fold change in the levels of TLR9 mRNA extracted from 3 × 10^6^ mouse peritoneal macrophages stimulated with 1 × 10^6^ *Giardia lamblia* virus (GLV)–containing *Giardia* trophozoites for various periods (0, 2, 4, 6, 8, and 12 h). **(B)** RT-qPCR analysis of the relative fold change in the levels of TLR9 mRNA extracted from 3 × 10^6^ mouse peritoneal macrophages stimulated with gDNA (3 μg, extracted from 1 × 10^6^ GLV-free *Giardia* trophozoites), 1 × 10^6^ GLV-free *Giardia* trophozoites, 1 × 10^6^ GLV-containing *Giardia* trophozoites, or CpG ODN 1668 (5 μM/mL). The TLR9 mRNA level was normalized to that of β-actin. **(C)** The secretion levels of IL-6, TNF-α, and IL-12 p40 in cell culture supernatants were measured by ELISA. Data are expressed as means ± SD from three separate experiments. ns, no significant difference, *p < 0.05, **p < 0.01, ***p < 0.001.

To explore the role of TLR9 in *Giardia* infection, we first assessed TLR9 gene expression levels at different time points (0, 2, 4, 6, 8, and 12 h) using reverse transcriptase (RT)–quantitative PCR (qPCR). The results showed that PMφs incubated with *G. duodenalis* exhibited significantly enhanced transcription levels of TLR9 within 12 h compared to the control PMφs, peaking at 8 h and then decreasing (Figure 1A). To then address whether TLR9 activation was indeed due to stimulation by *Giardia* gDNA and not GLV or any other factor, we compared TLR9 mRNA levels among several stimulation groups, including a positive control group. RT-qPCR analysis indicated that TLR9 transcript levels in WT PMφs were increased to varying degrees, and no significant difference was found between PMφs stimulated with GLV-free and GLV-containing *Giardia* trophozoites (Figure 1B). Furthermore, to determine the effect of TLR9 on cytokine production by innate immune cells, we measured the production of IL-6, TNF-α, and IL-12 p40 in siTLR9-treated and WT PMφs. The results showed that incubation with *G. duodenalis* trophozoites increased the secretion levels of IL-6, TNF-α, and IL-12 p40 in WT PMφs; however, compared to WT PMφs, the secretion of these cytokines was markedly decreased in siTLR9-treated PMφs stimulated with CpG ODN 1668 or *G. duodenalis* trophozoites, but remained largely unchanged in the LPS-stimulated groups. In addition, WT PMφs stimulated with GLV-containing *Giardia* trophozoites exhibited significantly higher levels of cytokine secretion compared with stimulation with GLV-free *Giardia* trophozoites (Figure 1C and Supplementary Figure 1B).

### *G. duodenalis* Trophozoites Induced Cytokine Secretion in PMφs by Activating the p38 and ERK Pathways via TLR9

**FIGURE 2:**
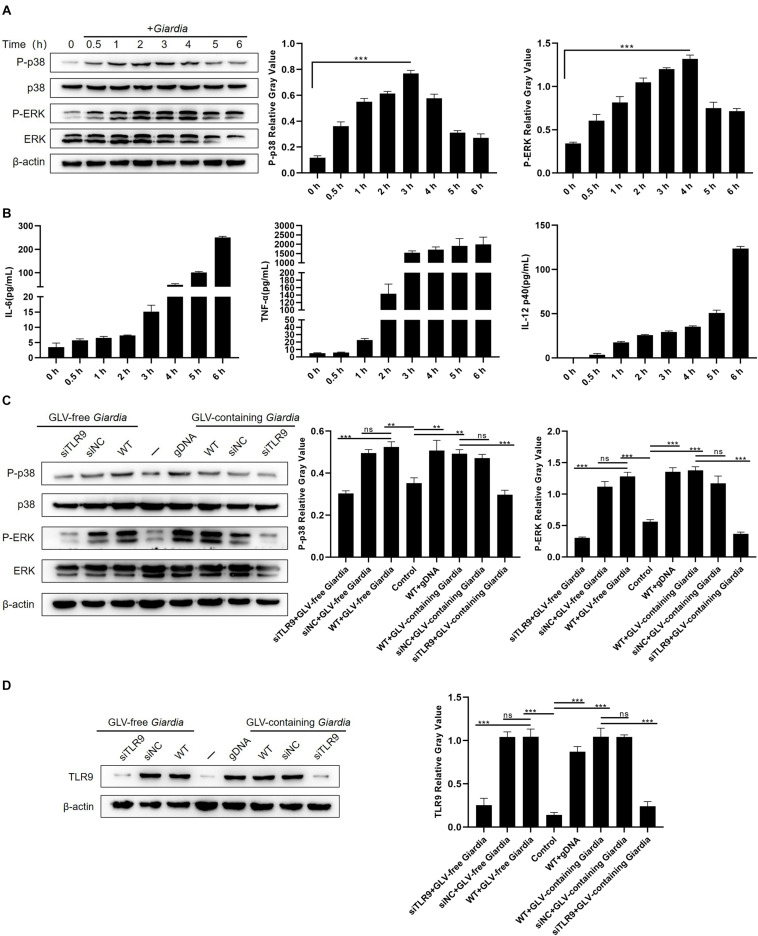
*Giardia duodenalis* trophozoites activated the p38 and ERK/MAPK signaling pathways *via* TLR9. **(A)** A total of 3 × 10^6^ wild-type (WT) mouse peritoneal macrophages were stimulated with 1 × 10^6^ *G*. *duodenalis* trophozoites for various periods (0–6 h) following which the phosphorylation levels of p38 and ERK were analyzed by Western blot. **(B)** The secretion levels of IL-6, TNF-α, and IL-12 p40 in cell culture supernatants were measured by ELISA. **(C)** Macrophages treated or not with small-interfering RNA (siRNA) targeting TLR9 (siTLR9) were stimulated with *Giardia lamblia* virus (GLV)–free or GLV-containing *Giardia* trophozoites for 3 h. **(D)** A total of 3 × 10^6^ WT macrophages pretreated or not with siTLR9 were incubated for 3 h with 1 × 10^6^ GLV-free *Giardia* trophozoites or 1 × 10^6^ GLV-containing *Giardia* trophozoites, following which TLR9 expression levels were analyzed by Western blot. Relative protein expression was quantified by densitometric analysis using β-actin as an internal reference. Data are expressed as means ± SD from three separate experiments. ns, no significant difference, *p < 0.05, **p < 0.01, ***p < 0.001.

To examine whether stimulation with *G. duodenalis* trophozoites could activate the p38 and ERK pathways in mouse macrophages, the phosphorylation levels of p38 and ERK in WT PMφs were measured by Western blot after stimulation with *G. duodenalis* trophozoites. The phosphorylation of p38 and ERK was increased within 6 h compared with that in control PMφs, with that of p38 peaking after 3 h and then decreasing and that of ERK peaking at 4 h followed by a decrease (Figure 2A). Additionally, to evaluate cytokine levels following *G. duodenalis* stimulation, we assessed the levels of secreted cytokines (IL-6, TNF-α, and IL-12 p40) in cell supernatants and found that production of these cytokines by WT PMφs showed a gradual increase (Figure 2B).

To estimate whether the *G. duodenalis* trophozoites–induced activation of the p38 and ERK pathways was mediated by TLR9, PMφs treated or not with siTLR9 were stimulated with GLV-free or GLV-containing *Giardia* trophozoites for 3 h at 37°C. Compared with WT or siNC-treated PMφs incubated with *G. duodenalis* trophozoites, the phosphorylation levels of p38 and ERK in siTLR9-treated PMφs incubated with *G. duodenalis* trophozoites were greatly decreased (Figure 2C). Furthermore, to assess the efficacy of siTLR9, we measured the TLR9 protein expression levels by Western blot in siTLR9-treated PMφs. The results showed that stimulated with LPS, CpG ODN 1668, GLV-free *Giardia* trophozoites, or GLV-containing *Giardia* trophozoites, protein expression levels of TLR9 significantly decreased in WT PMφs with TLR9-siRNA pretreatment compared with those in untreated PMφs (Figure 2D and Supplementary Figure 1A). These results suggested that *G. duodenalis* trophozoites–induced activation of the p38 and ERK signaling pathways was mediated *via* TLR9.

**FIGURE 3:**
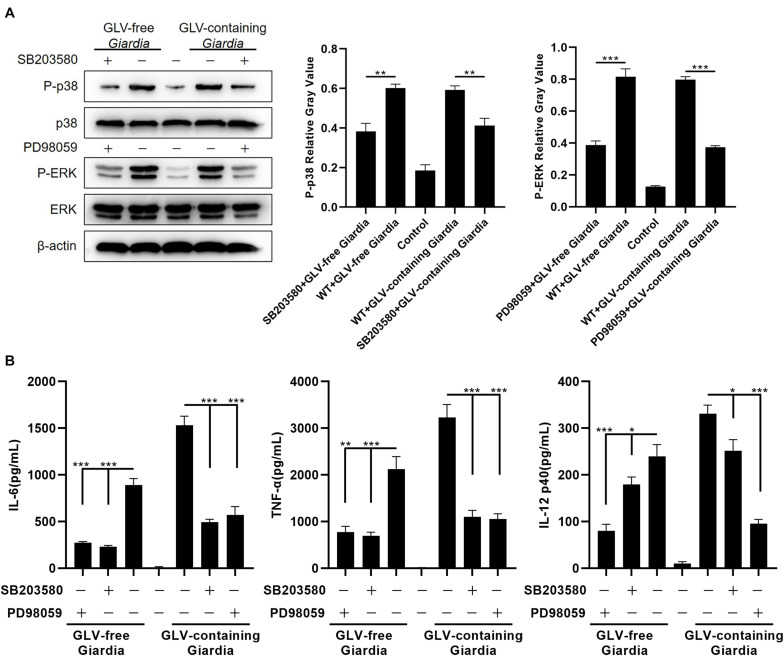
*Giardia duodenalis* trophozoites–induced cytokine production was disrupted by p38 and ERK inhibitor treatment. **(A)** A total of 3 × 10^6^ wild-type (WT) mouse peritoneal macrophages were pretreated for 30 min with the p38 inhibitor SB203580 (30 μM) or the ERK inhibitor PD98059 (40 μM) before stimulation with 1 × 10^6^ *Giardia lamblia* virus (GLV)–free *Giardia* trophozoites or 1 × 10^6^ GLV-containing *Giardia* trophozoites. The phosphorylation levels of p38 and ERK were subsequently analyzed by Western blot. Relative protein expression was quantified by densitometric analysis using β-actin as an internal reference. **(B)** The production of IL-6, TNF-α, and IL-12 p40 in cell supernatants was measured by ELISA. Data are expressed as means ± SD from three separate experiments. *p < 0.05, **p < 0.01, ***p < 0.001.

To evaluate the role of the p38 and ERK signaling pathways in modulating the production of IL-6, TNF-α, and IL-12 p40, PMφs were pretreated or not with the p38 inhibitor SB203580 or the ERK inhibitor PD98059 for 30 min at 37°C. Following incubation with GLV-free or GLV-containing *Giardia* trophozoites for 18 h, the bottom-layer cells were harvested for Western blotting, and the cell supernatants were used for the measurement of cytokine concentrations by ELISA. Western blot analysis demonstrated that p38 and ERK phosphorylation levels were markedly diminished in PMφs pretreated with the inhibitors compared with those in untreated PMφs (Figure 3A). Moreover, the *G. duodenalis* trophozoites–induced secretion of IL-6, TNF-α, and IL-12 p40 was significantly decreased in PMφs pretreated with the inhibitors compared with that of untreated PMφs (Figure 3B). These data demonstrated that the p38 and ERK pathways were activated *via* TLR9, leading to increased secretion of IL-6, TNF-α, and IL-12 p40 in PMφs exposed to *G. duodenalis* trophozoites.

### *G. duodenalis* Trophozoites Reduced Cytokine Production by Activating the AKT Pathway

**FIGURE 4:**
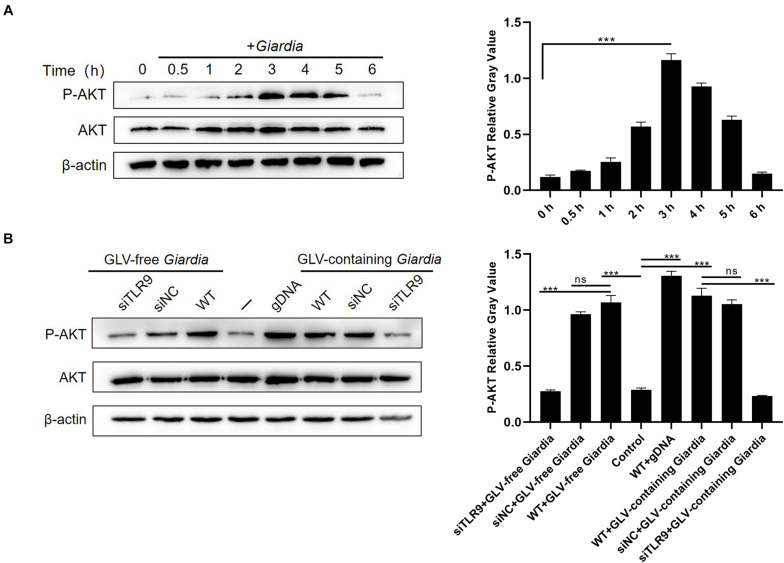
*Giardia duodenalis* trophozoites induced the phosphorylation of AKT *via* TLR9. **(A)** A total of 3 × 10^6^ wild-type (WT) mouse peritoneal macrophages were stimulated with 1 × 10^6^ *G. duodenalis* trophozoites for various periods (0–6 h) after which the phosphorylation level of AKT was analyzed by Western blot. **(B)** A total of 3 × 10^6^ macrophages treated or not with siRNA targeting TLR9 were stimulated with 1 × 10^6^ *Giardia lamblia* virus (GLV)–free *Giardia* trophozoites or 1 × 10^6^ GLV-containing *Giardia* trophozoites for 3 h. Data are expressed as means ± SD from three separate experiments. ns, no significant difference, *p < 0.05, **p < 0.01, ***p < 0.001.

To investigate whether stimulation with *G. duodenalis* trophozoites could activate the AKT pathway in mouse macrophages, AKT phosphorylation levels in *G. duodenalis* trophozoites–incubated PMφs were determined by Western blot. The results showed that AKT phosphorylation levels were first increased compared with that of control PMφs, peaking at 3 h, and then decreased to baseline levels at 6 h (Figure 4A).

To assess whether the *G. duodenalis* trophozoites–induced activation of the AKT pathway was mediated *via* TLR9, AKT phosphorylation levels were measured in PMφs treated or not with siTLR9 and stimulated with GLV-free or GLV-containing *Giardia* trophozoites for 3 h at 37°C. AKT phosphorylation levels were significantly reduced in the siTLR9-treated groups with *G. duodenalis* trophozoites compared with those in stimulated WT PMφs. These results suggested that *G. duodenalis* trophozoites activated the AKT signaling pathway through TLR9 (Figure 4B).

**FIGURE 5:**
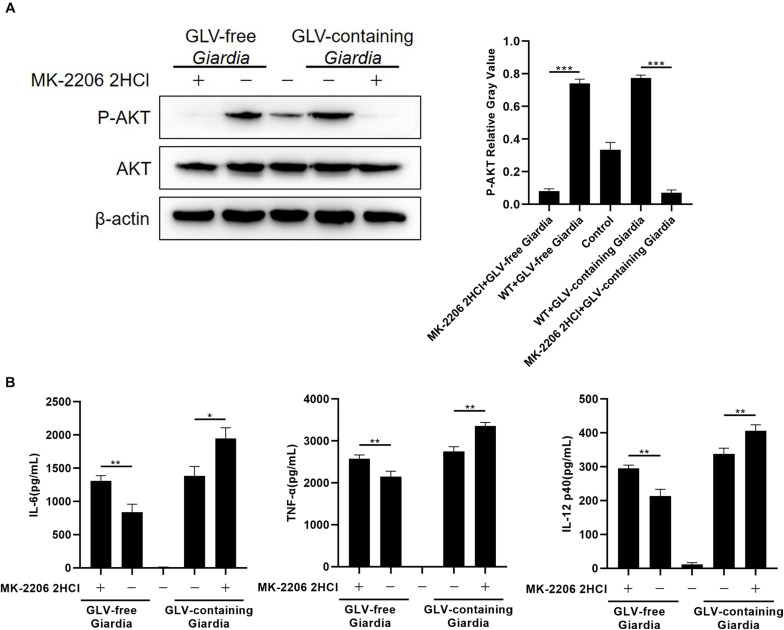
*Giardia duodenalis* trophozoites suppressed cytokine production by activating the AKT signaling pathway. **(A)** A total of 3 × 10^6^ wild-type (WT) mouse peritoneal macrophages were pretreated for 30 min with the AKT inhibitor MK-2206 2HCl (5 μM) before stimulation with *Giardia lamblia* virus (GLV)–free or GLV-containing *Giardia* trophozoites. The AKT phosphorylation level was subsequently analyzed by Western blot. Relative protein expression was quantified by densitometric analysis using β-actin as an internal reference. **(B)** The secretion levels of IL-6, TNF-α, and IL-12 p40 in cell supernatants were measured by ELISA. Data are expressed as means ± SD from three separate experiments. *p < 0.05, **p < 0.01, ***p < 0.001.

To explore the role of the AKT signaling pathway in regulating the production of IL-6, TNF-α, and IL-12 p40, PMφs were pretreated or not with the AKT inhibitor MK-2206 2HCl for 30 min at 37°C. Following coincubation with GLV-free or GLV-containing *Giardia* trophozoites for 18 h, the bottom-layer cells were harvested for Western blotting, whereas the cell supernatants were used for the determination of cytokine concentrations by ELISA. Western blot analysis demonstrated that pretreatment with the AKT inhibitor significantly reduced AKT phosphorylation levels (Figure 5A). Furthermore, compared with untreated PMφs, the *G. duodenalis* trophozoites–induced secretion of IL-6, TNF-α, and IL-12 p40 was increased to varying degrees in PMφs pretreated with the AKT inhibitor (Figure 5B). These data indicated that *G. duodenalis* reduced the secretion of these cytokines in PMφs by activating the AKT signaling pathway.

### *G. duodenalis* Trophozoites Induced the Nuclear Translocation of NF-κB p65 in SiTLR9-Treated and WT PMφs

**FIGURE 6:**
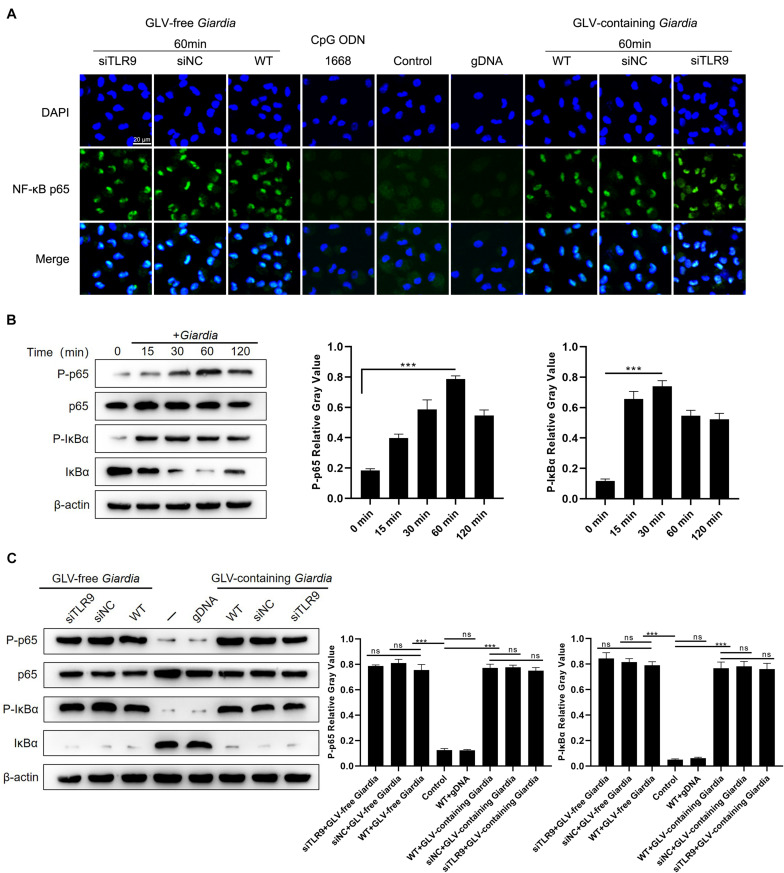
*Giardia duodenalis* trophozoites activated the NF-κB signaling pathway by inducing NF-κB p65 nuclear translocation. **(A)** Laser confocal microscopic images showing the facilitation of the nuclear translocation of NF-κB p65 by *G*. *duodenalis* in wild-type (WT) mouse macrophages treated or not with small-interfering RNA (siRNA) targeting TLR9 (siTLR9). **(B)** A total of 3 × 10^6^ WT macrophages were stimulated with 1 × 10^6^ *G. duodenalis* trophozoites for various periods (0–120 min) after which the phosphorylation levels of NF-κB p65 and IκBα were analyzed by Western blot. **(C)** A total of 3 × 10^6^ WT macrophages treated or not with siTLR9 were stimulated with 1 × 10^6^ *Giardia lamblia* virus (GLV)–free *Giardia* trophozoites or 1 × 10^6^ GLV-containing *Giardia* trophozoites for 60 min. Relative protein expression was quantified by densitometric analysis using β-actin as an internal reference. Data are expressed as means ± SD from three separate experiments. ns, no significant difference, *p < 0.05, **p < 0.01, ***p < 0.001.

To determine the effects of *G. duodenalis* trophozoites on NF-κB activation, immunofluorescence staining and Western blot were used to detect the localization and expression of NF-κB p65, respectively. Translocation of NF-κB p65 into the nucleus of PMφs following incubation with *G. duodenalis* trophozoites was visualized by laser confocal microscopy (Figure 6A). Western blot analysis showed that, in WT PMφs incubated with *G. duodenalis* trophozoites, NF-κB p65 phosphorylation peaked after 60 min and then gradually decreased, whereas the phosphorylation of IκBα peaked at 30 min and then gradually decreased (Figure 6B). Laser confocal microscopy revealed that after stimulation with *G. duodenalis* trophozoites for 60 min, NF-κB p65 could be seen in the nuclei of both siTLR9-treated and WT PMφs, whereas no nuclear translocation was detected in WT PMφs without *G. duodenalis* trophozoite stimulation as well as in group stimulated with CpG ODN 1668 or gDNA (Figure 6A). To further investigate whether the *G. duodenalis* trophozoites–induced activation of NF-κB was mediated *via* TLR9, PMφs treated or not with siTLR9 were stimulated with GLV-free or GLV-containing *Giardia* trophozoites for 60 min at 37°C. No differences in NF-κB p65 or IκBα phosphorylation levels were found between siTLR9-treated and WT PMφs (Figure 6C), confirming the results obtained by immunofluorescence staining. Combined, these results indicated that the *G. duodenalis* trophozoites–induced activation of NF-κB was not mediated *via* TLR9.

## Discussion

*Giardia duodenalis* colonization of the intestinal tract of the host damages the host intestinal mucosa (Roxström-Lindquist et al., 2005; Chen et al., 2013). This leads to macrophage recruitment in the intestine, which triggers the innate immune response, followed by the secretion of proinflammatory cytokines to defend against *Giardia* infection (Roxström-Lindquist et al., 2005; Maloney et al., 2015). Meanwhile, adaptive immune responses are initiated, including the mobilization and recruitment of dendritic cells (DCs) for antigen processing and presentation (Rescigno et al., 2001; Roxström-Lindquist et al., 2006), thereby further enhancing the host immune response to *G. duodenalis*. Throughout this process, macrophages play a pivotal role. It has been demonstrated that macrophages can intake *G. duodenalis* trophozoites and gather them in the lamina propria of the small intestine (Belosevic and Daniels, 1992; Maloney et al., 2015). TLRs are crucial components of innate immunity, mediating inflammatory responses to defend against invading pathogens. Located on the cell membrane and in intracellular endosomes, TLRs can monitor and recognize a range of PAMPs derived from bacteria, parasites, viruses, and fungi. Importantly, studies have revealed the functions and mechanisms associated with the innate immune responses mediated by TLR2 and TLR4 during *Giardia* infection (Lee et al., 2014; Li et al., 2017; Serradell et al., 2019; Zhao et al., 2021). Additionally, it has been shown that the *Plasmodium falciparum*–derived metabolite, hemozoin, can activate TLR9 in host macrophages and DCs, leading to the massive secretion of proinflammatory factors and chemokines (Kawai and Akira, 2009; Kumar et al., 2009). Here, we found that both GLV-free and GLV-containing *Giardia* trophozoites could significantly enhance TLR9 gene expression in WT mouse PMφs. Notably, our study was performed on mouse macrophages infected with *G. duodenalis*, whereas *Giardia muris* might be a better option for establishing a model of *Giardia* infection in mice (Roberts-Thomson et al., 1976; Stevens and Roberts-Thompson, 1978); moreover, *Giardia*-induced activation of TLRs, including TLR9, may differ among *Giardia* species (Koh et al., 2013; Bhargava et al., 2015). Given that the disparities between strains were not examined in this study, further studies are warranted to investigate the differences between *Giardia* strains in eliciting TLR-mediated host innate immune responses. In addition, *G. duodenalis* is the only known zoonotic protozoan parasite among *Giardia* species, and studies on the involvement of immune cells in host–pathogen interactions are commonly performed using human peripheral blood mononuclear cells (Cheng et al., 2017; Cromarty et al., 2019), which may be a better alternative than mouse macrophages for investigating the mechanisms involved in immune cell defenses against *G. duodenalis* infection. Relevant studies involving human immune cells and various animal models are needed to further validate the current findings.

The proinflammatory cytokines TNF-α and IL-6 are essential for the elimination and early control of giardiasis in mice (Zhou et al., 2003, 2007; Li et al., 2004). Interestingly, TNF-α deficiency does not affect the mechanisms involved in host defenses against *Giardia* infection, which includes the generation of IgA, the proliferation of mast cells, and the secretion of IL-6 or IL-4, suggesting that TNF-α does not exert its effects through these mechanisms mentioned previously in control of *Giardia*, but through other effector responses downstream of it (Zhou et al., 2007). IL-6 serves a vital function in the eradication of *Giardia* in infected mice (Zhou et al., 2003). For example, IL-6 deficiency can significantly affect the immune response to *G. duodenalis* (Bienz et al., 2003), and IL-6 can regulate B-cell maturation and induce a shift of antibody type to IgA in response to *Giardia* infection (Ekdahl and Andersson, 2005). Additionally, mast cells can rapidly produce IL-6 to control *Giardia* infection in mice (Li et al., 2004). *Giardia* induces inflammatory responses that involve blood platelets and the release of IL-6 and TNF-α in patients infected with *G. duodenalis* (Matowicka-Karna et al., 2009). Additionally, bone marrow–derived DCs coincubated with *Giardia* extracts and TLR ligands, including CpG DNA, exhibited increased secretion of IL-10 and diminished secretion of IL-12 (Kamda and Singer, 2009). The results of the present study indicated that, following stimulation with *G. duodenalis* trophozoites or CpG ODN 1668, the secretion levels of IL-6, TNF-α, and IL-12 p40 were significantly higher in WT macrophages than in macrophages treated with siTLR9, but were largely unchanged in the LPS-stimulated groups, suggesting that siTLR9 treatment specifically silenced TLR9 without blocking the activation of macrophages by another pathway. These data indicated that the *G. duodenalis–*induced secretion of proinflammatory cytokines in macrophages was in part TLR9-dependent. Furthermore, IL-6 and TNF-α are known to be involved in the elimination and early control of *Giardia* infection. In the present study, we further found that, with *G. duodenalis* trophozoite stimulation, the secretion levels of IL-6 and TNF-α were significantly lower in siTLR9-treated mouse macrophages than in WT mouse macrophages, suggesting that activated TLR9 not only has a role in the regulation of cytokine secretion by the host in the early stages of infection but also plays a host-protective role. Importantly, GLV-containing *Giardia* trophozoites elicited significantly greater production of proinflammatory cytokines, including IL-6, TNF-α, and IL-12 p40, than GLV-free *Giardia* trophozoites, suggesting GLV may be a factor that contributed to this difference. And it has been reported that the dsRNA genome of *Leishmaniavirus* is recognized by the host endosomal TLR3, which triggers the secretion of proinflammatory cytokines and chemokines (Ives et al., 2011; Zangger et al., 2014). In the present study, TLR9 in mouse macrophages could recognize CpG DNA motifs, which may exist in *G. duodenalis* rather than the dsRNA genome of GLV. That the TLR3 of the host recognizes dsRNA viral genomes, such as that of the *Leishmania* RNA virus, suggests that the GLV dsRNA genome in GLV-containing *Giardia* trophozoites may be recognized by TLR3 in mouse macrophages, thus causing the host to initiate a more intense inflammatory response. In addition, another potential explanation for the difference in host inflammatory response induced by *G. duodenalis* is that GLV-free and GLV-containing *Giardia* trophozoites are derived from different *G. duodenalis* isolates, rather than the effect of GLV. And studies have found that *G. duodenalis* assemblage B, including GS isolate, produces more inflammatory responses both *in vitro* and *in vivo* compared to *G. duodenalis* assemblage A (Hanevik et al., 2007; Bénéré et al., 2012; Lee et al., 2012).

Mitogen-activated protein kinase signaling cascades are crucial for monitoring the host’s immune responses to infection, regulating the transcription of numerous proinflammatory cytokine-related genes through the phosphorylation of transcription factors and the promotion of chromatin remodeling (Kirk et al., 2020). The expression of TLR2, TLR4, and TLR9 was reported to be significantly upregulated in HeLa cells stimulated with *Trichomonas vaginalis* in a p38 signaling pathway-dependent manner (Chang et al., 2006). *G. duodenalis* GS excretory/secretory products contain factors that prompt HT-29 cells to secrete IL-8 through activating the p38 and ERK1/2 signaling pathways (Lee et al., 2012). Our findings revealed that the phosphorylation levels of p38 and ERK/MAPKs in siTLR9-treated macrophages were significantly decreased compared with those of WT macrophages. Moreover, stimulation of mouse macrophages with GLV-free and GLV-containing *Giardia* trophozoites, respectively, resulted in different cytokines secretion, and higher secretion levels of IL-6, TNF-α, and IL-12 p40 were observed in macrophages with GLV-containing *Giardia* trophozoite stimulation compared to GLV-free *Giardia* trophozoites; however, such secretion of *G. duodenalis–*induced cytokines was abolished by pretreatment with p38 and ERK inhibitors. Corroborating the findings of our present study, the *G. duodenalis–*induced secretion of IL-6, TNF-α, and IL-12 p40 was decreased in siTLR9-treated mouse macrophages and WT mouse macrophages pretreated with p38 and ERK inhibitors, implying that the TLR9–p38/ERK signaling pathways activated by *G. duodenalis* may contribute to host defenses against *Giardia* infection. These findings suggested that the *G. duodenalis–*induced secretion of proinflammatory cytokines in macrophages was achieved *via* TLR9-mediated activation of p38 and ERK signaling.

AKT is a serine/threonine-protein kinase with a pivotal part in the regulation of cell metabolism, survival, and proliferation. In macrophage-mediated innate immunity, AKT regulates the expression and production of proinflammatory cytokines (Lee et al., 2011). Studies have shown that, under chronic stress, the levels of phospho-AKT are lower in TLR9-deficient macrophages than in WT macrophages (Xiang et al., 2015). Additionally, *G. duodenalis* exposure was reported to reduce the secretion of proinflammatory cytokines in a TLR2-mediated, AKT-dependent manner (Li et al., 2017). In our study, the phosphorylation of AKT in siTLR9-treated macrophages was significantly decreased compared with that in WT macrophages. Furthermore, pretreatment with an AKT inhibitor increased the secretion levels of IL-6, TNF-α, and IL-12 p40 in mouse macrophages stimulated with GLV-free or GLV-containing *Giardia* trophozoites, implying that both GLV-free and GLV-containing *Giardia* trophozoites can attenuate the secretion levels of these cytokines in mouse macrophages *via* the AKT signaling pathway. These data suggested that *G. duodenalis* trophozoites can downregulate cytokine secretion by mouse macrophages in an AKT-dependent manner. However, the differences between these results and those obtained with the TLR9 silencing assay and p38/ERK inhibition experiments suggest that the positive regulatory effects associated with the p38 and ERK pathways were more important than the negative regulatory effects related to the AKT pathway for the TLR9-mediated innate immune response against *Giardia* infection in mouse macrophages.

The NF-κB transcriptional complex is known to be a key regulator of cellular stress responses and immune responses to infection (Ashall et al., 2009). NF-κB activity in the nucleus is controlled by IκB proteins, which provide transient or dynamic regulation through their stimuli-responsive degradation and resynthesis (Adelaja and Hoffmann, 2019). Studies have suggested that the transition from p50 to p65 heterodimers to p50 homodimer in NF-κB may be involved in inflammation (Lawrence et al., 2001; Ashall et al., 2009). Additionally, as a TLR9 ligand, CpG DNA triggers the production of immune mediators by immune cells through TLR9-mediated signal transduction, which, in turn, leads to NF-κB activation (Xu et al., 2003). It has been reported that *G. duodenalis* may target NF-κB in a differential manner to modulate the inflammatory functions of macrophages (Faria et al., 2020). In our study, the immunofluorescence assay showed that both GLV-free and GLV-containing *Giardia* trophozoites could induce NF-κB p65 accumulation in the nucleus in both siTLR9-treated and WT mouse macrophages. Further, Western blot analysis showed that the phosphorylation levels of p65 and IκBα in siTLR9-treated macrophages were similar to those of WT macrophages. Additionally, GLV-free and GLV-containing *Giardia* trophozoites exerted similar effects on activating the NF-κB signaling pathway. These results indicated that *G. duodenalis* can activate NF-κB p65 signaling in a TLR9-independent manner in mouse macrophages.

Collectively, our results showed that *G. duodenalis* can induce the production of proinflammatory cytokines through the TLR9-mediated activation of the p38 and ERK signaling pathways in mouse PMφs, whereas GLV-containing *Giardia* trophozoites can enhance cytokine secretion in mouse macrophages to a greater extent than GLV-free *Giardia* trophozoites. Furthermore, *G. duodenalis–*induced activation of TLR9–p38/ERK signaling enhanced immune defense responses, thus forming a host-protective barrier. Our study complements those on TLR-mediated innate immune responses of host cells against *Giardia* infection and provides potential molecular targets for the further development of novel strategies for the treatment of giardiasis.

## Data Availability Statement

The original contributions presented in the study are included in the article/Supplementary Material, further inquiries can be directed to the corresponding author.

## Ethics Statement

The animal study was reviewed and approved by the Animal Welfare and Research Ethics Committee at Jilin University (IACUC Permit Number: 20160612).

## Author Contributions

XP, XL, and PZ drafted the manuscript and analyzed the data. XP, KY, LC, and PG planned and performed the experiments. XP, XL, ML, ZZ, NZ, XW, and PG designed the experiments. XZ, JL, and PG provided guidance and support and revised the manuscript. All authors contributed to the article and approved the submitted version.

## Conflict of Interest

The authors declare that the research was conducted in the absence of any commercial or financial relationships that could be construed as a potential conflict of interest.
