# Supplementary material for: Giardia duodenalis Induces Proinflammatory Cytokine Production in Mouse Macrophages via TLR9-Mediated p38 and ERK Signaling Pathways
Source: Front Cell Dev Biol. 2021 Jul 15;9:694675. doi: 10.3389/fcell.2021.694675 (PMC8319647; doi:10.3389/fcell.2021.694675)
Supplement: Supplementary file 1 [file Data_Sheet_1.PDF]

Supplementary Material

1 Supplementary Data

1.1 Uncropped western-blotting images are arranged in order below.

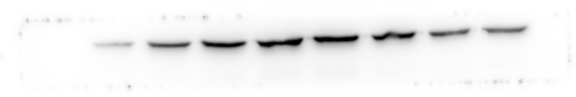

Figure 2A P-p38

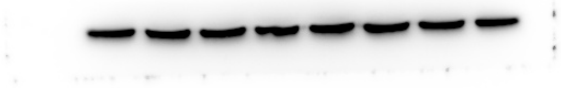

Figure 2A p38

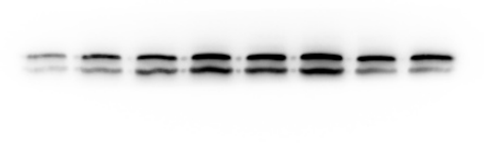

Figure 2A P-ERK

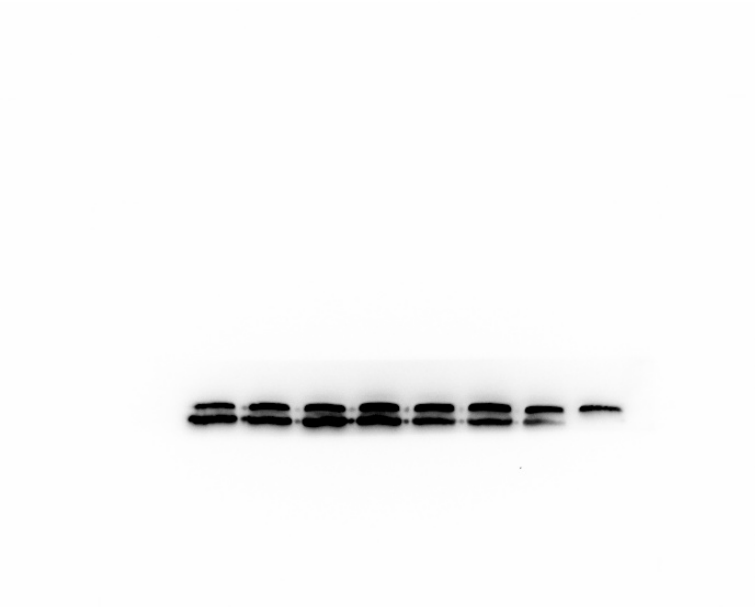

Figure 2A ERK

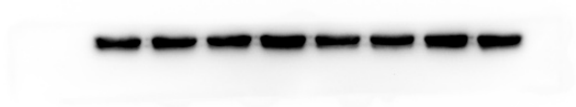

Figure 2A β-actin

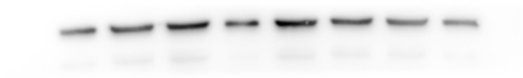

Figure 2C P-p38

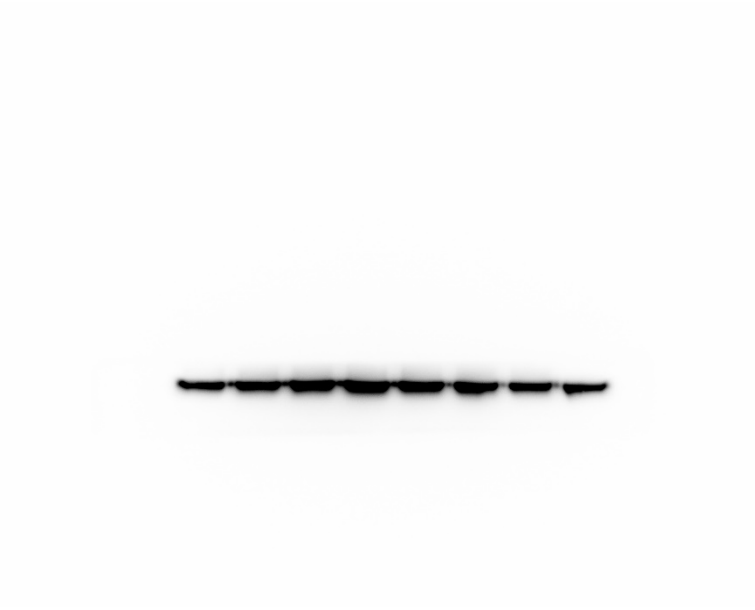

Figure 2C p38

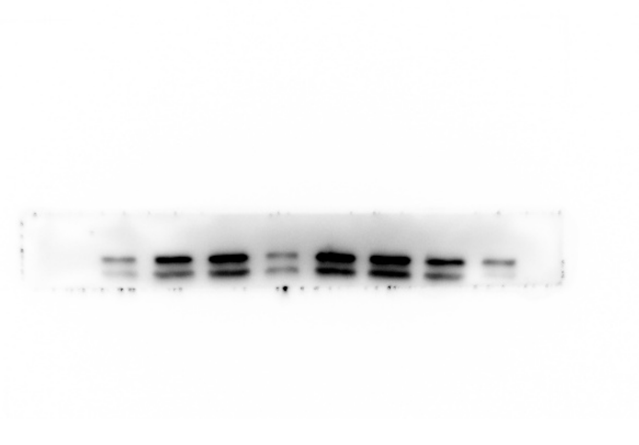

Figure 2C P-ERK

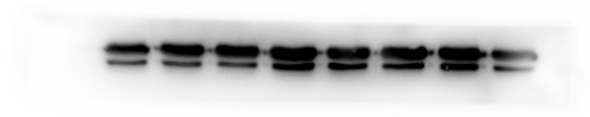

Figure 2C ERK

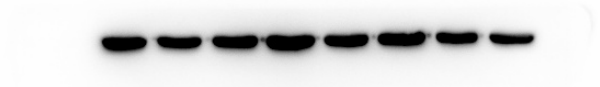

Figure 2C β-actin

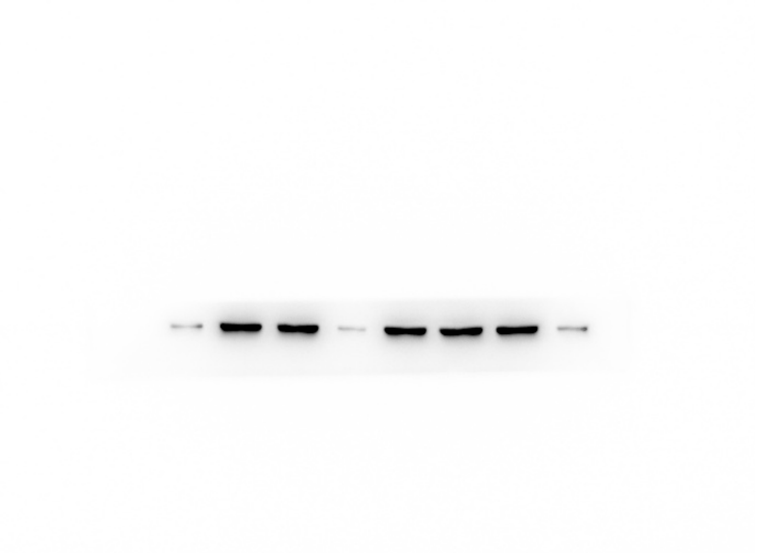

Figure 2D TLR9

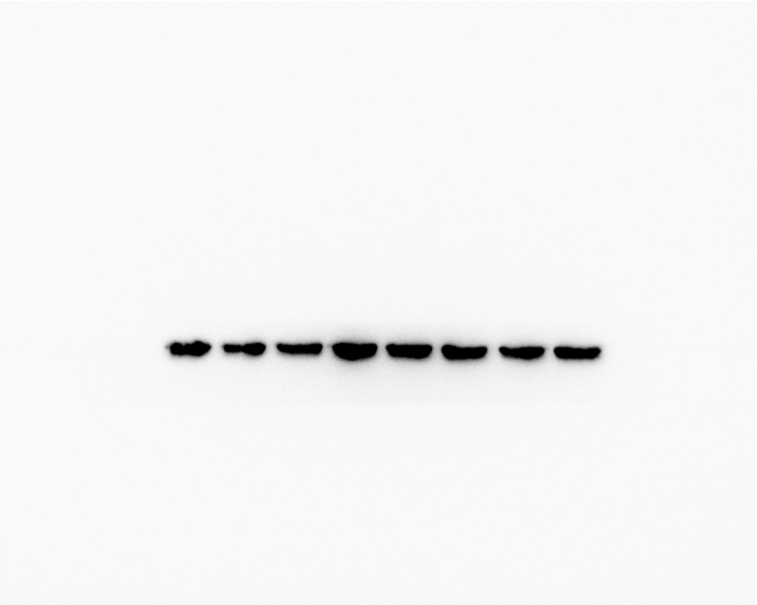

Figure 2D β-actin

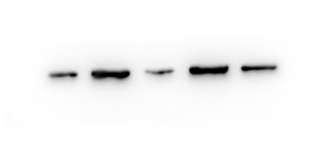

Figure 3A P-p38

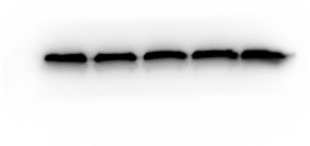

Figure 3A p38

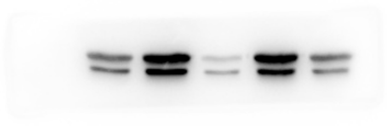

Figure 3A P-ERK

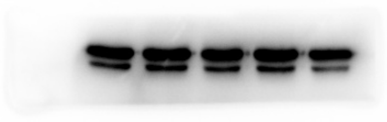

Figure 3A ERK

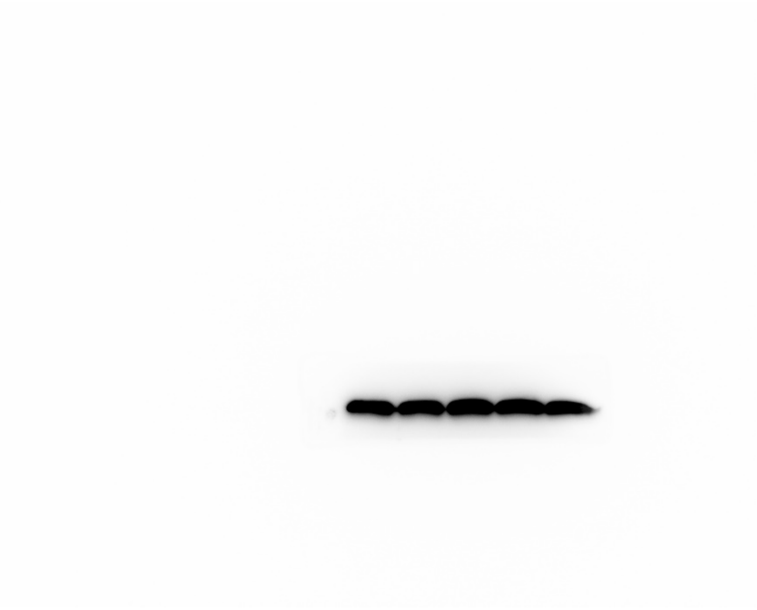

Figure 3A β-actin

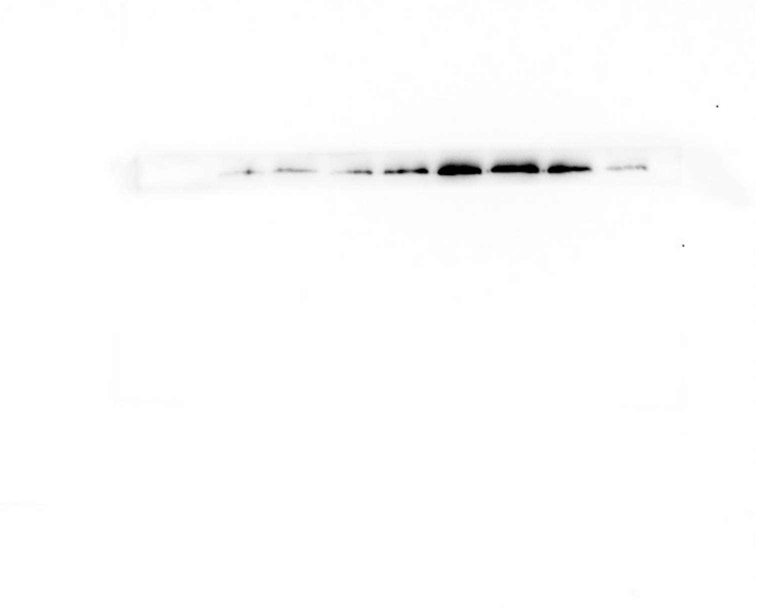

Figure 4A P-AKT

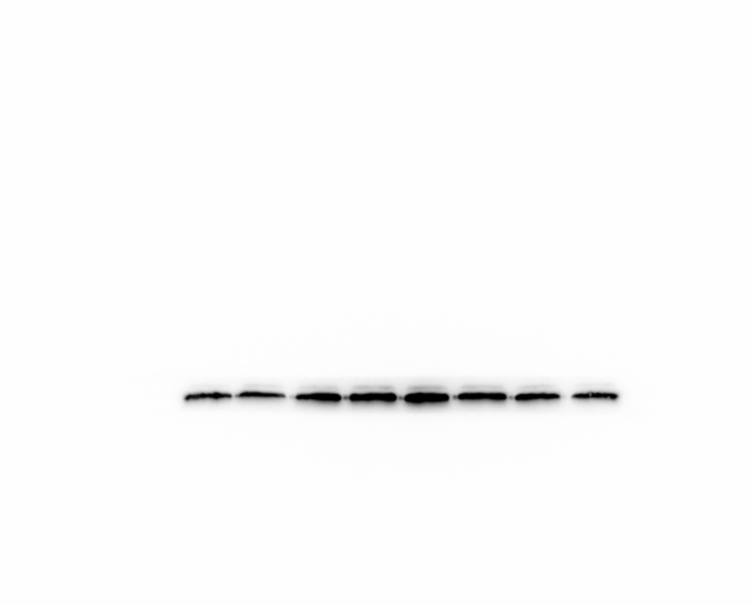

Figure 4A AKT

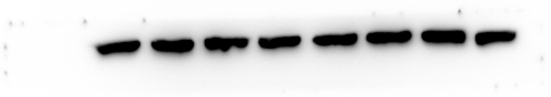

Figure 4A β-actin

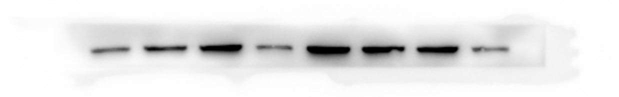

Figure 4B P-AKT

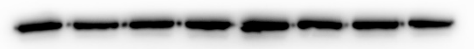

Figure 4B AKT

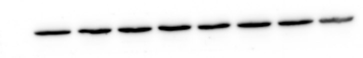

Figure 4B β-actin

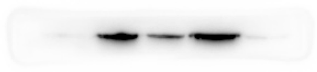

Figure 5A P-AKT

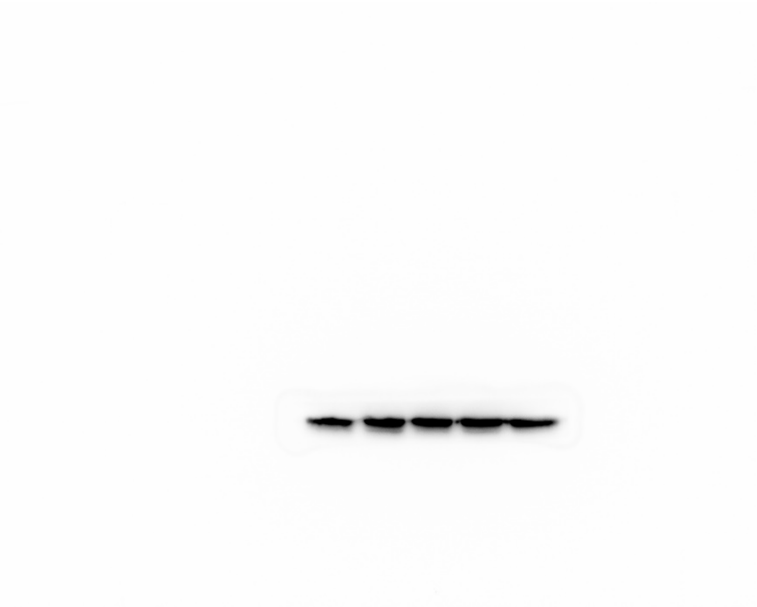

Figure 5A AKT

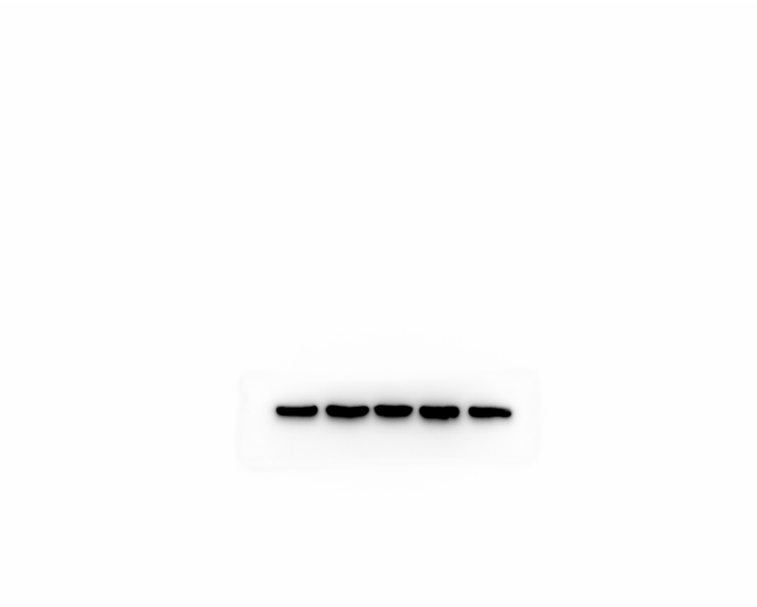

Figure 5A β-actin

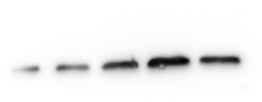

Figure 6B P-p65

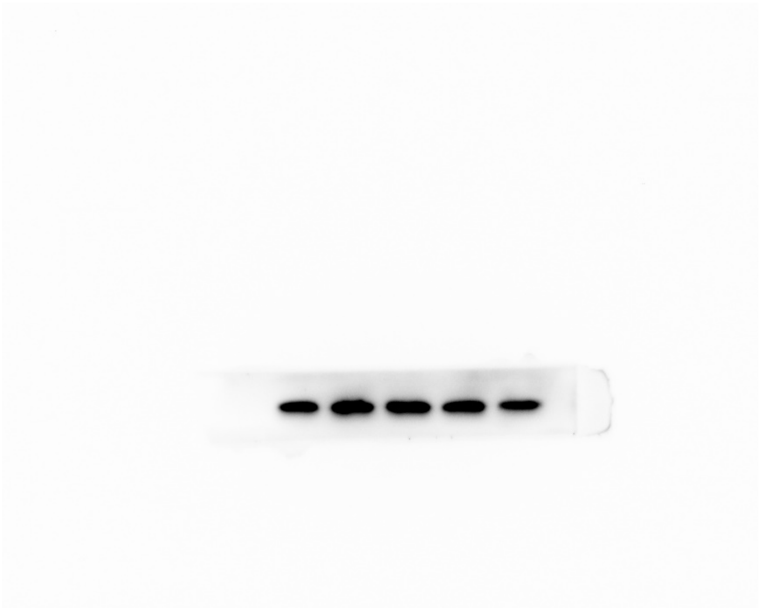

Figure 6B p65

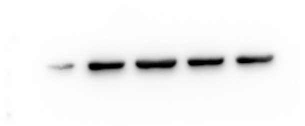

Figure 6B P-IκBα

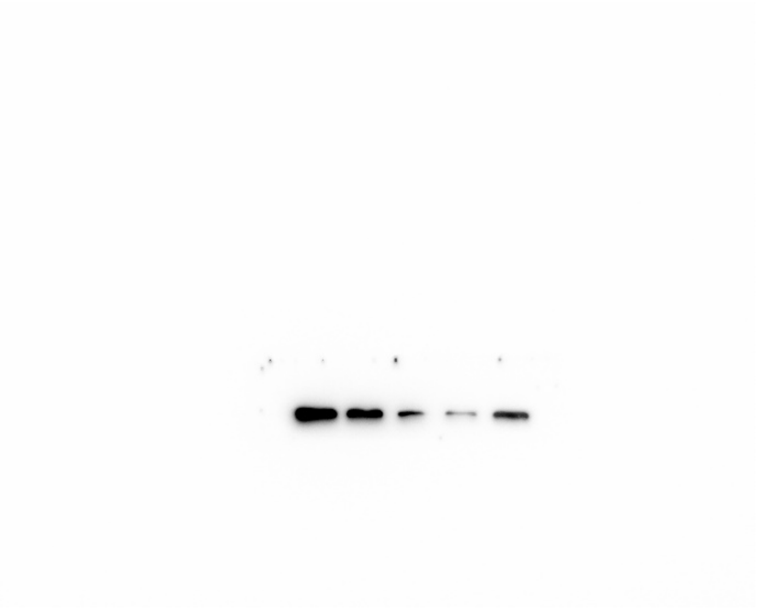

Figure 6B IκBα

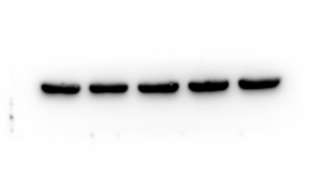

Figure 6B β-actin

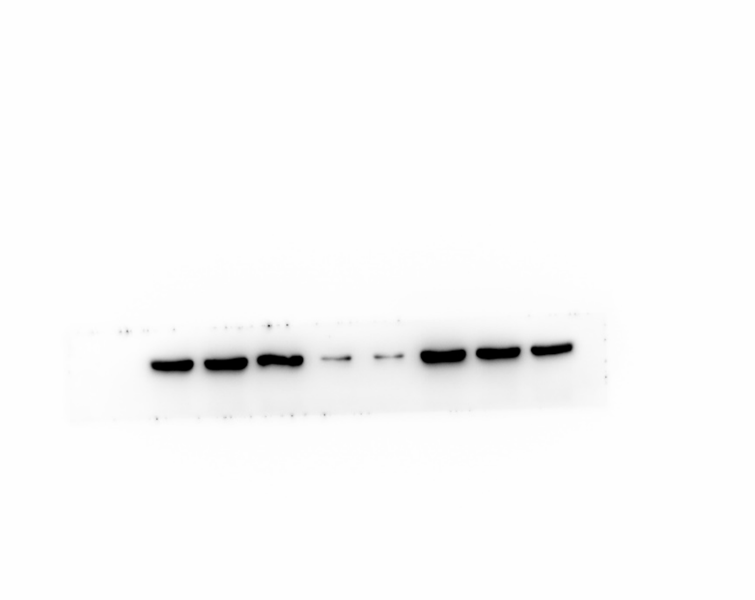

Figure 6C P-p65

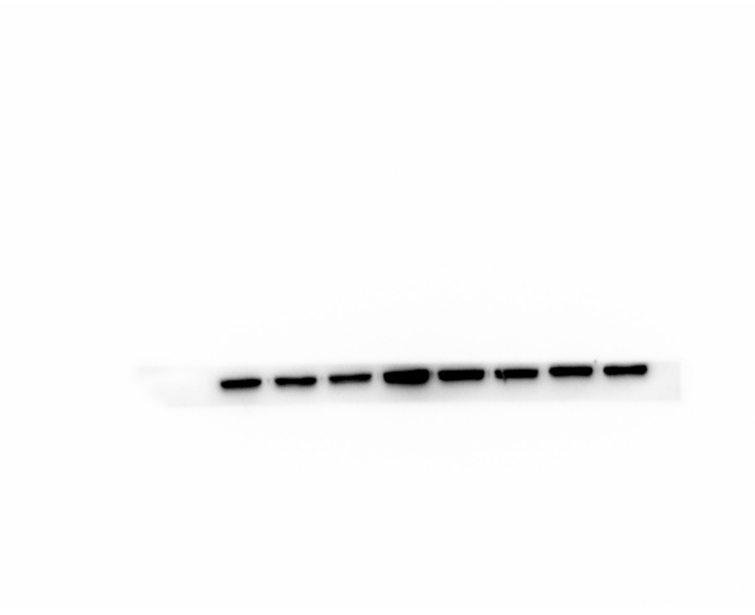

Figure 6C p65

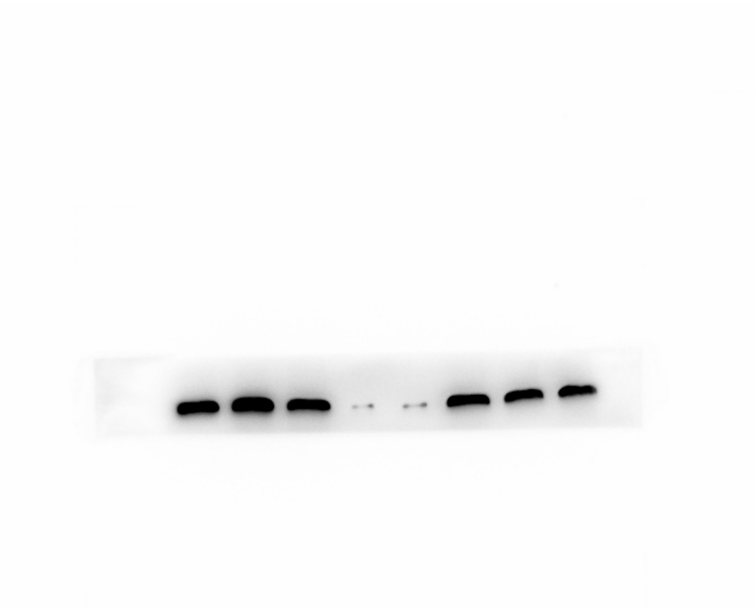

Figure 6C P-IκBα

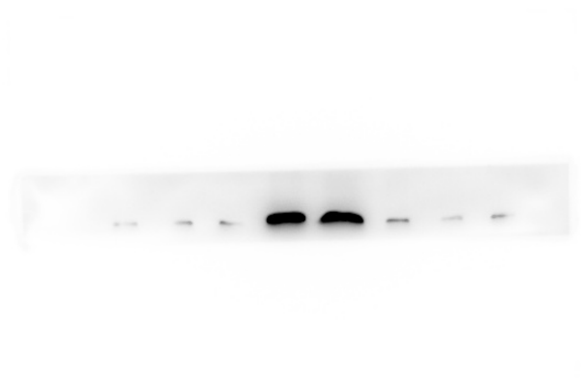

Figure 6C IκBα

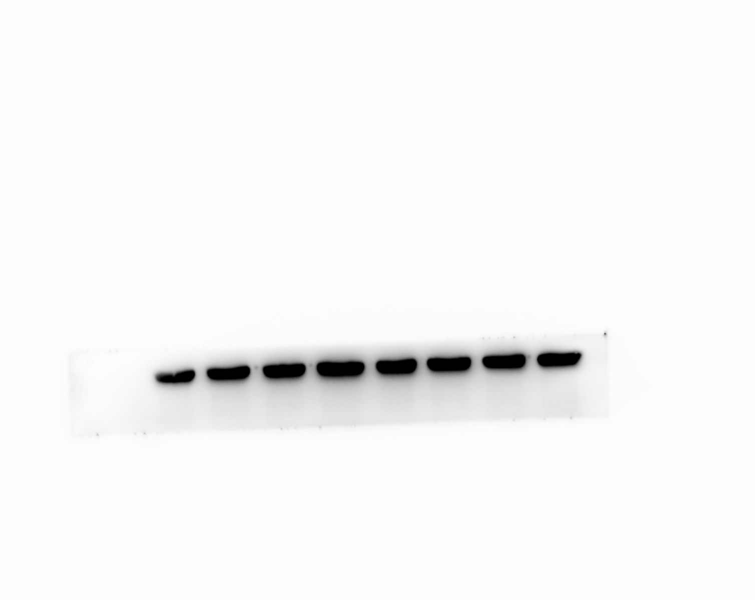

Figure 6C β-actin

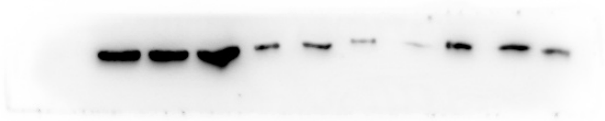

Figure S1 TLR9

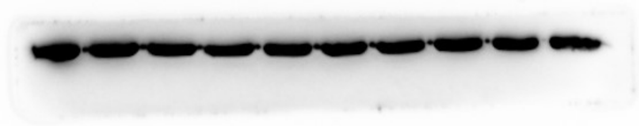

Figure S1 β-actin

1.2 Electron micrograph observation of *Giardia* virus-like particles

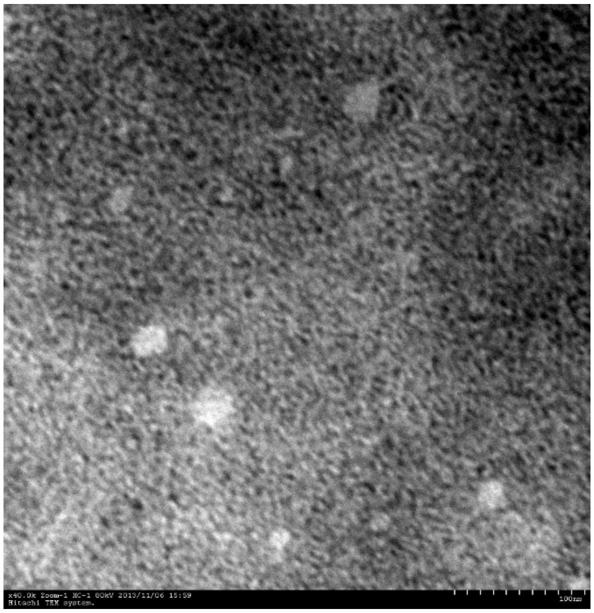

Transmission electron microscopical observation of extracts of GLV-free *Giardia* trophozoites

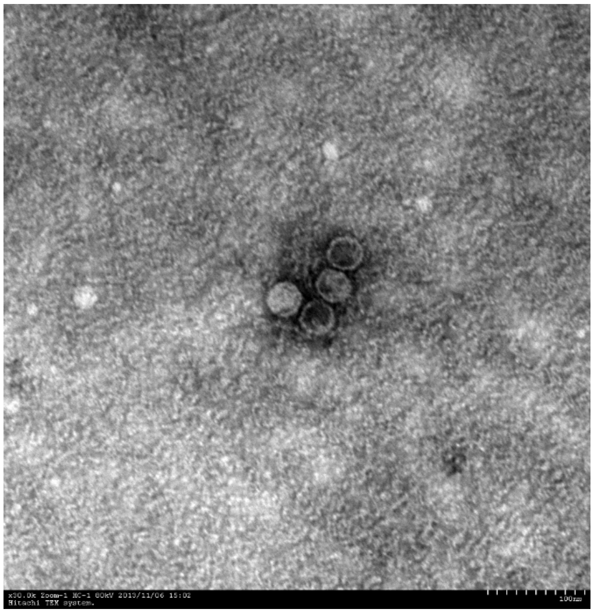

Transmission electron microscopical observation of extracts of GLV-containing *Giardia* trophozoites

2 Supplementary Figures and Tables

2.1 Supplementary Figures

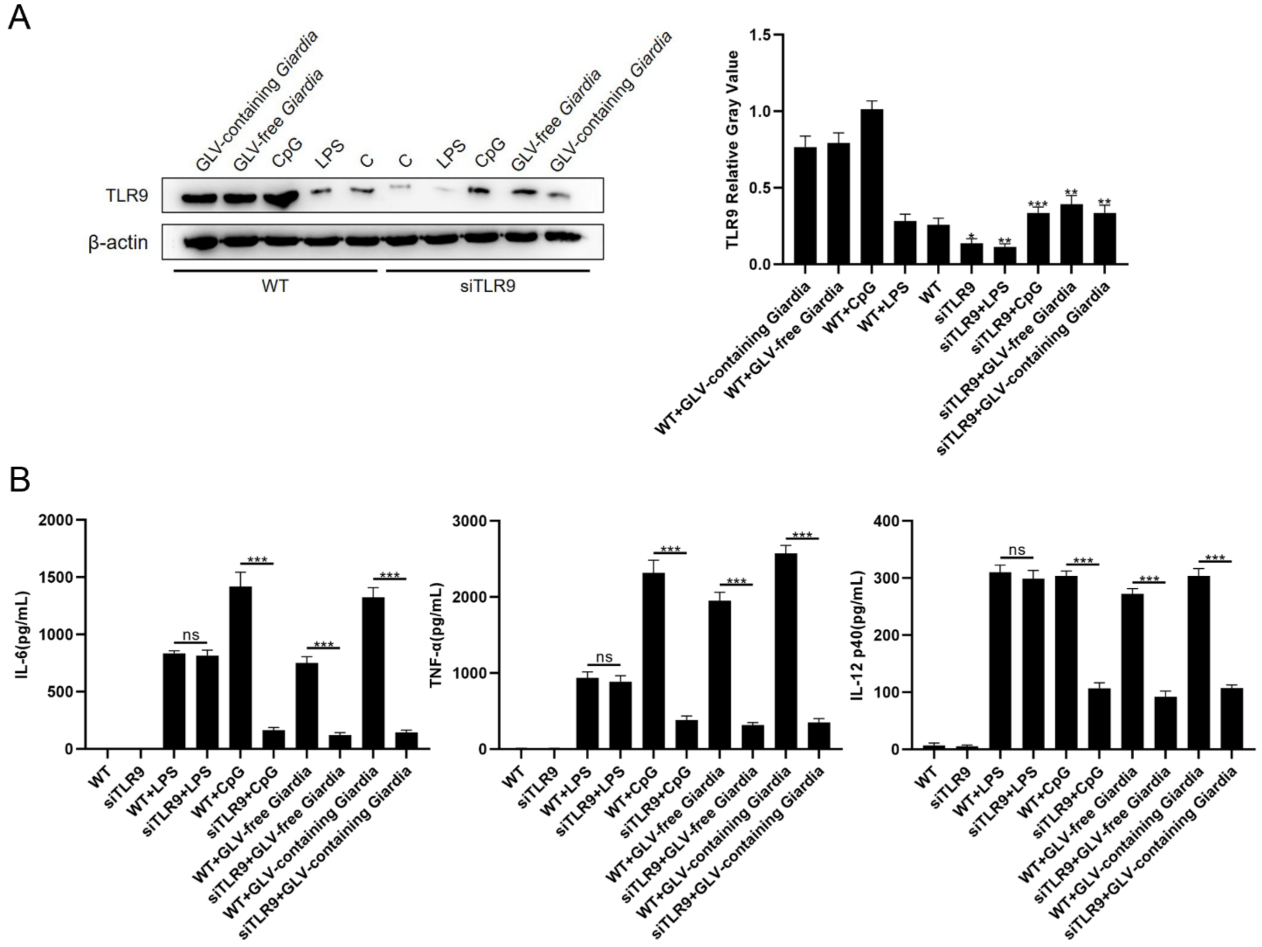

Supplementary Figure 1 | *Giardia duodenalis* trophozoites induced cytokine production in a TLR9-dependent manner. **(A)** A total of 3 × 10^6 WT macrophages pretreated or not with siTLR9 were incubated for 3 h with LPS (100 ng/mL), CpG ODN 1668 (5 μM/mL), 1 × 10^6 *Giardia lamblia* virus (GLV)-free *Giardia* trophozoites or 1 × 10^6 GLV-containing *Giardia* trophozoites, following which TLR9 expression levels were analyzed by western blot. Relative protein expression was quantified by densitometric analysis using β-actin as an internal reference. **(B)** The secretion levels of IL-6, TNF-α, and IL-12 p40 in cell culture supernatants were measured by ELISA. Data are expressed as means ± SD from three separate experiments. ns, no significant difference, *$p < 0.05$, **$p < 0.01$, ***$p < 0.001$.
