# Supplementary figures and images for: Giardia duodenalis Induces Proinflammatory Cytokine Production in Mouse Macrophages via TLR9-Mediated p38 and ERK Signaling Pathways
Source: Front Cell Dev Biol. 2021 Jul 15;9:694675. doi: 10.3389/fcell.2021.694675 (PMC8319647; doi:10.3389/fcell.2021.694675)

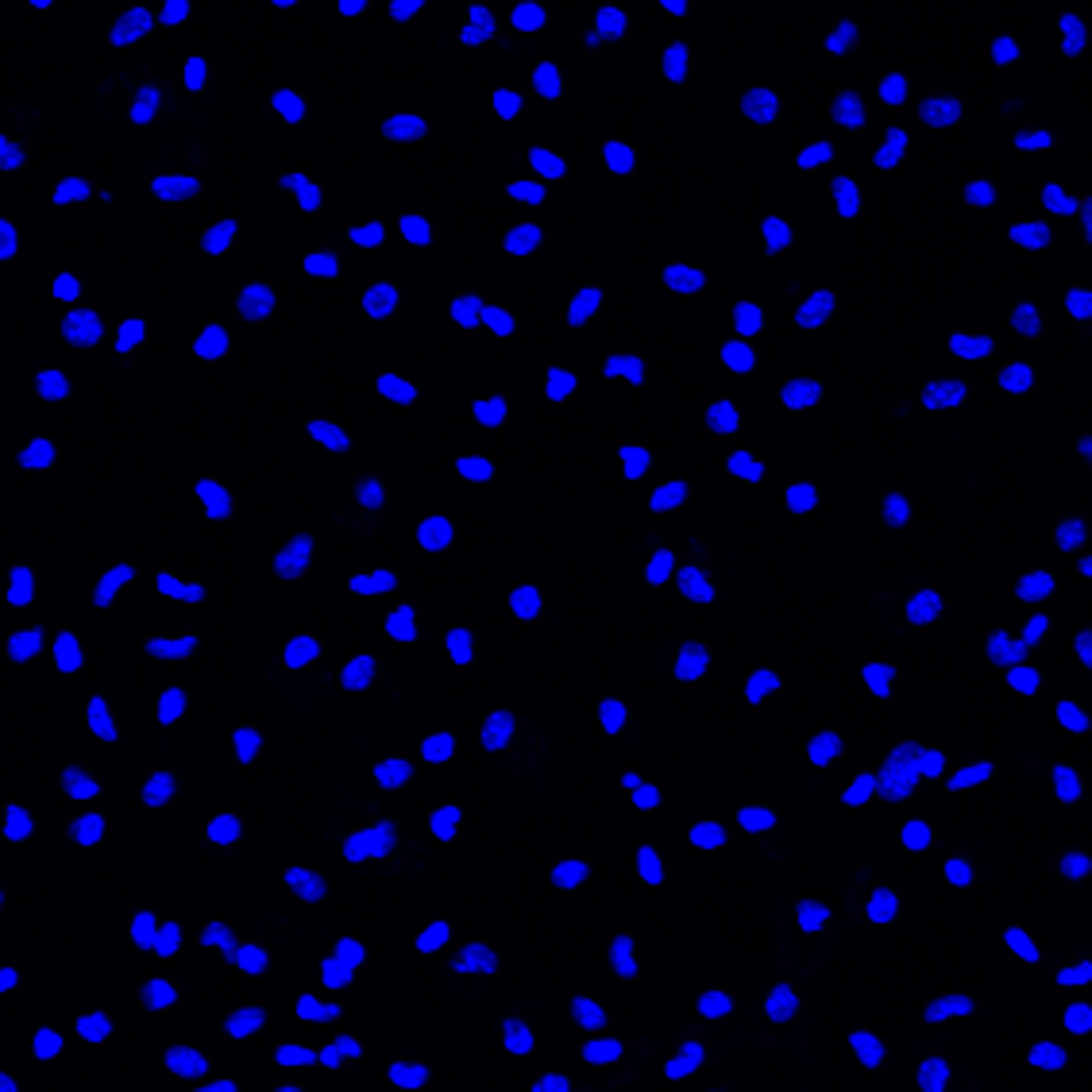

Supplement: Supplementary file 2 [file Data_Sheet_2.zip › Immunofluorescence images of the control groups/Control-DAPI.tif]

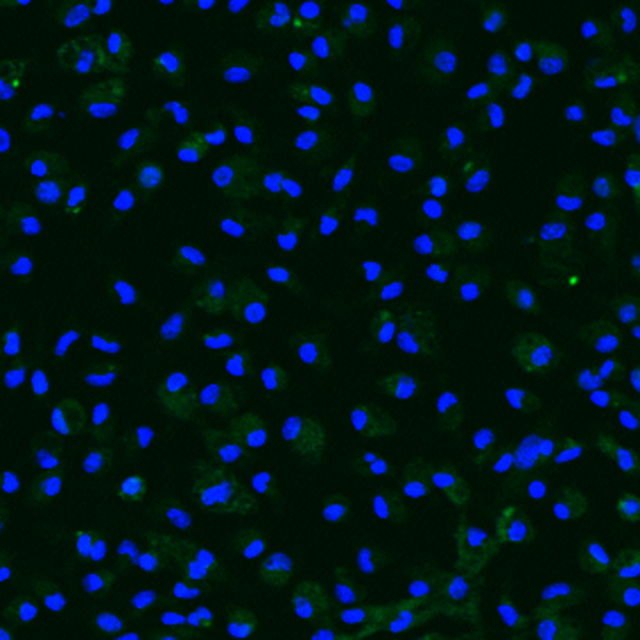

Supplement: Supplementary file 2 [file Data_Sheet_2.zip › Immunofluorescence images of the control groups/Control-Merge.tif]

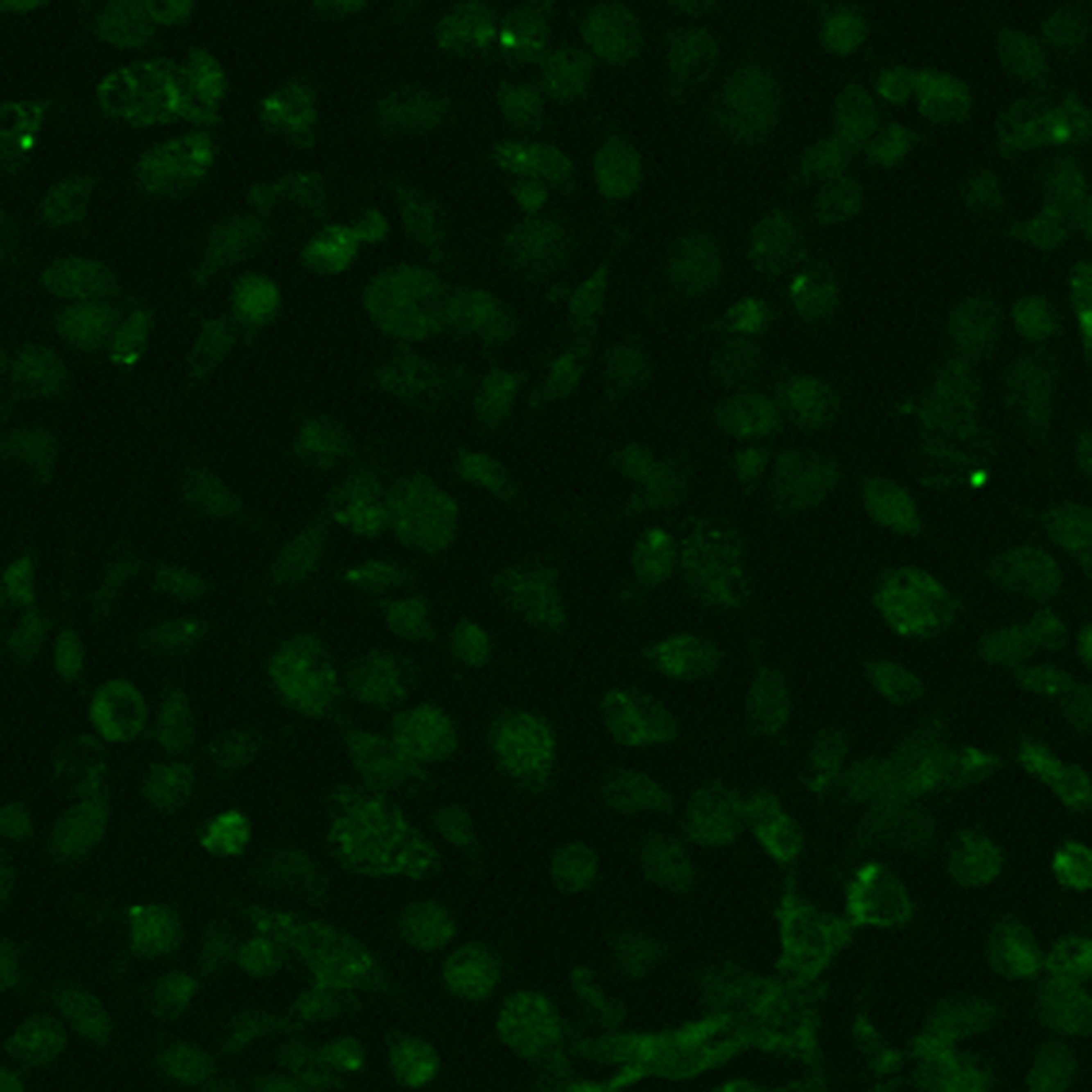

Supplement: Supplementary file 2 [file Data_Sheet_2.zip › Immunofluorescence images of the control groups/Control-p65.tif]

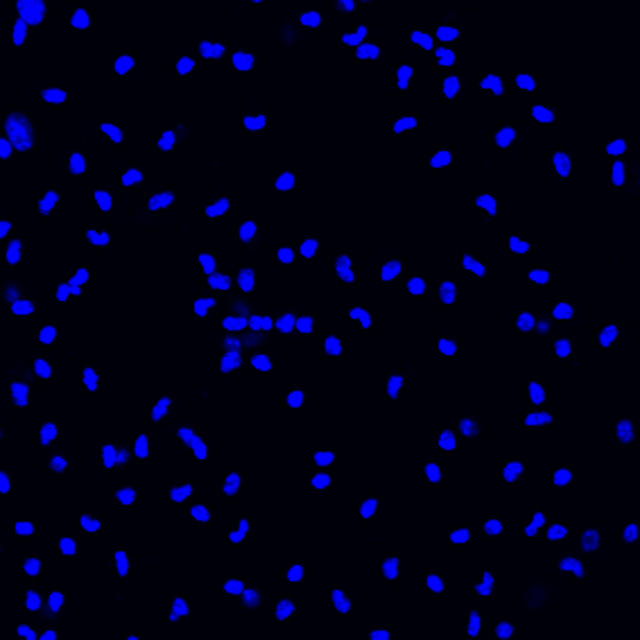

Supplement: Supplementary file 2 [file Data_Sheet_2.zip › Immunofluorescence images of the control groups/CpG ODN 1668-DAPI.tif]

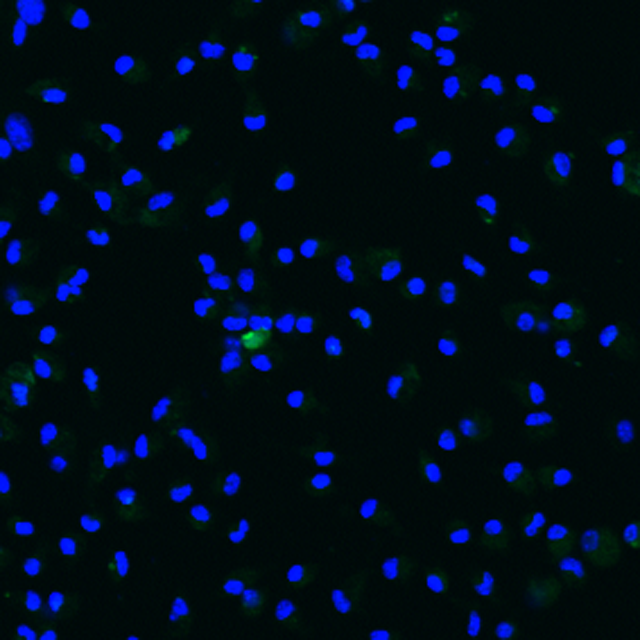

Supplement: Supplementary file 2 [file Data_Sheet_2.zip › Immunofluorescence images of the control groups/CpG ODN 1668-Merge.tif]

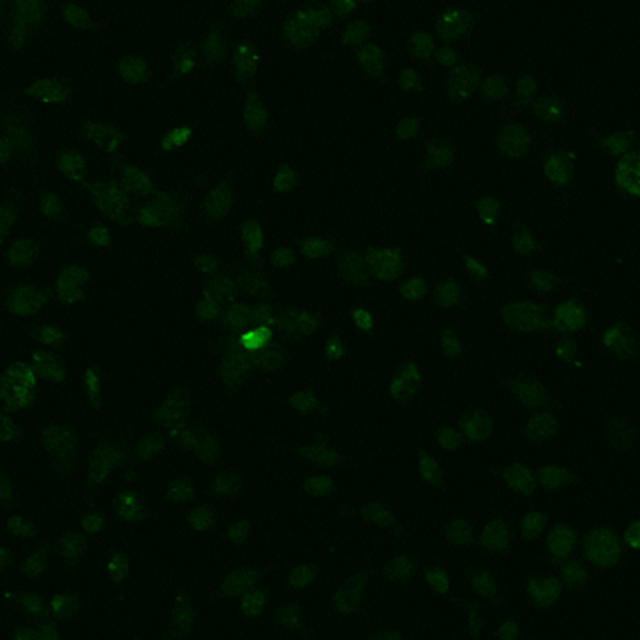

Supplement: Supplementary file 2 [file Data_Sheet_2.zip › Immunofluorescence images of the control groups/CpG ODN 1668-p65.tif]

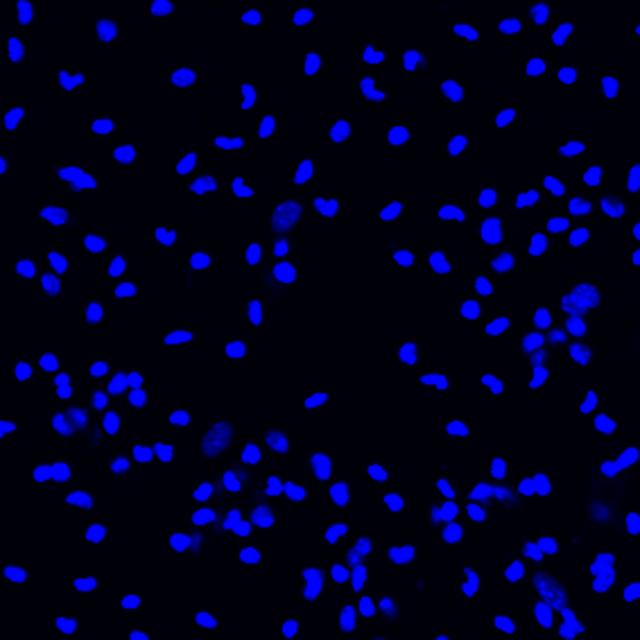

Supplement: Supplementary file 2 [file Data_Sheet_2.zip › Immunofluorescence images of the control groups/gDNA-DAPI.tif]

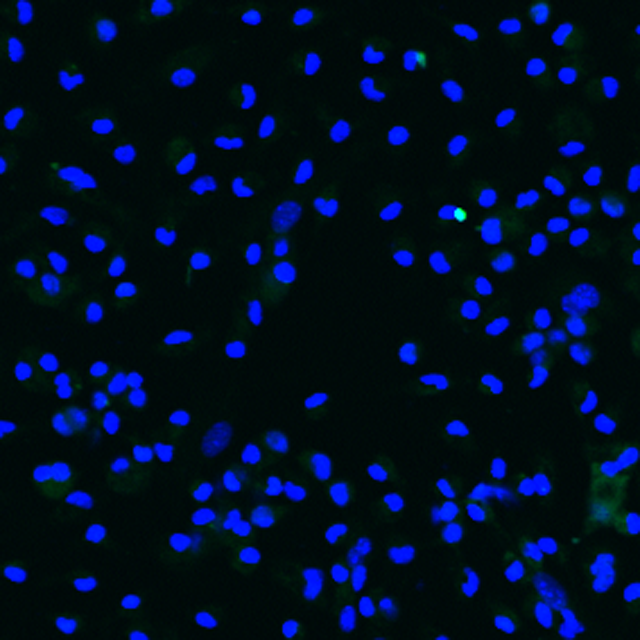

Supplement: Supplementary file 2 [file Data_Sheet_2.zip › Immunofluorescence images of the control groups/gDNA-Merge.tif]

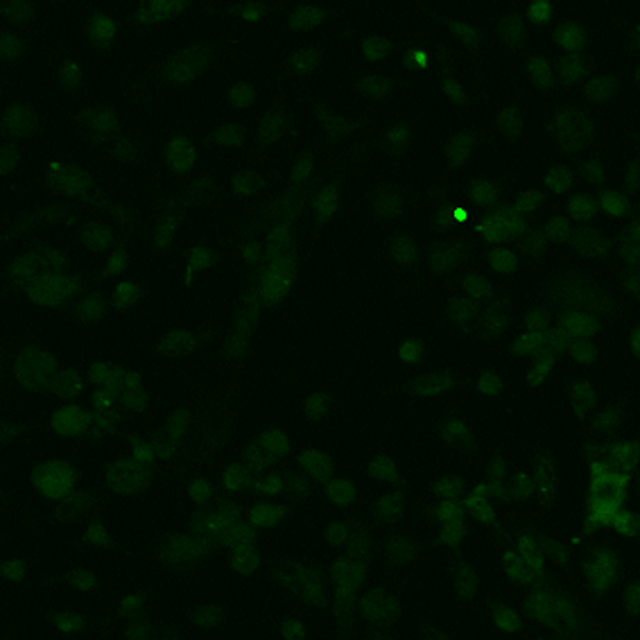

Supplement: Supplementary file 2 [file Data_Sheet_2.zip › Immunofluorescence images of the control groups/gDNA-p65.tif]
